# Integrative analysis of the metabolome and transcriptome reveals the mechanism of flower color and antioxidant capacity in three *Syringa* cultivars

**DOI:** 10.3389/fpls.2026.1840931

**Published:** 2026-07-03

**Authors:** Hailiang Zhang, Xiaofei Wang, Siting Wang, Zhiying Yang, Huayang Li, Meng Li, Xiaoyu Zhang, Xuekai Gao, YueBo Sun, Kezhong Zhang, Ruidong Han, Qingzhu Zhao, Shuhua Yang

**Affiliations:** 1 Weifang Academy of Agricultural Sciences, Weifang, China; 2 Key Laboratory of Biology and Genetic Improvement of Flower Crops (North China), Ministry of Agriculture and Rural Affairs, State Key Laboratory of Vegetable Biobreeding, Institute of Vegetables and Flowers, Chinese Academy of Agricultural Sciences, Beijing, China; 3 College of Landscape Architecture, Beijing University of Agriculture, Beijing, China; 4 Northern Agriculture and Livestock Husbandry Technology Innovation Center, Inner Mongolia Zhongnong North Agriculture and Animal Husbandry Technology Co., Ltd., Hohhot, China; 5 Weifang Vocational College, Weifang, China

**Keywords:** antioxidant, Color, Flavonoids, Metabolic flux partitioning, *Syringa vulgaris*

## Abstract

Lilac plants (*Syringa* spp.) are extensively valued in landscaping and human health for their colorful cultivars and significantly medicinal and edible properties. The current works mainly focus on the anthocyanidin-induced pigmentation or the flavonoid-driven antioxidant activity, respectively. Nevertheless, the genetic and metabolic panorama of flavonoids, especially the metabolic flux partitioning of anthocyanidin and other flavonoids, remains ambiguous in the *Syringa vulgari*s plants. In this study, integrating phenotypic, RNA sequencing, and metabolomic data, this study delineates the metabolic flux partitioning of flavonoids underlying floral pigmentation and antioxidant capacity across three *S. vulgaris* cultivars (blue, purple, and white). A core regulatory module of seven genes governs the biosynthesis of four flavonoid metabolites (kaempferol-3,7-diglucoside, kaempferol-3-sophorose-7-glucoside, kaempferol-3-rutinoside, and rutin) and two anthocyanidin metabolites (cyanidin-4-rutinoside and delphinidin-3-rutinoside), while shikimate acyltransferase gene (*SvHCT-4*) acted as a pivotal node directing flux toward cyanidin-4-rutinoside and delphinidin-3-rutinoside accumulation. These flavonoids and anthocyanidins in *S. vulgari*s cultivars orchestrated stage-specific modulation of floral pigmentation and differentially enhanced the scavenging capacities of 2,2-Diphenyl-2-picrylhydrazyl radical (DPPH·) and hydroxyl radical (OH·). *SvHCT-4* functions as a critical metabolic branch point governing metabolic flux partitioning from non-anthocyanin flavonoids to anthocyanin biosynthesis in the blue and purple cultivars of *S. vulgaris*, thereby enhancing floral pigmentation and antioxidant activity. The findings not only provided valuable insights into the intricate regulatory networks governing floral pigmentation and antioxidant capacity but also offered potential targets for future genetic engineering and breeding efforts aimed at enhancing these desirable traits in horticultural crops.

## Introduction

Color is a key trait in plant development, classification, and evolution (Yamasaki et al., 1996). Flower pigmentation is primarily determined by the composition of six major anthocyanidins including pelargonidin, cyanidin, delphinidin, peonidin, petunidin, and malvidin in plant (Forkmann, 1991; Lepiniec et al., 2006; Qi et al., 2023). In *Malus*, red color is governed by the anthocyanins-to-flavonoids ratio in the fruits (Han et al., 2023). Furthermore, the spatial distribution and structural modifications of anthocyanidins modulate color transformation of flowers through sugar glycosidic linkages in ornamental flowers (Bueno et al., 2012; Grotewold, 2006; Houghton et al., 2021; Manolescu et al., 2019). On the other hand, flavonoids from edible plants exhibit well-documented therapeutic benefits, chiefly via antioxidant mechanisms, and are widely utilized as dietary bioactives to support health outcomes across diverse populations (Hanganu et al., 2021; Kim and Shin, 2012; Zhang et al., 2009; Zhou et al., 2013). Flavonoids scavenge free radicals and reduce oxidative stress in edible flowers (Jakubczyk et al., 2022; Yiğit et al., 2022). Flavonoids as well as anthocyanins from a conserved metabolic pathway synergistically regulate plant functions like tissue pigmentation and antioxidant capacity. Peonidin and cyanidin cooperatively modulate floral pigmentation in *Hydrangea macrophylla* and *Carthamus tinctorius* (Peng et al., 2021; Wang et al., 2021). Insertion of retrotransposons in Ruby1 orchestrates cold-induced anthocyanin biosynthesis in blood oranges by integrating environmental signal transduction and metabolic flux reprogramming of the entire flavonoid pathway, thereby coupling pigmentation phenotype with adaptive antioxidant functions (Butelli et al., 2012). While flavonoids and anthocyanins are well-established as key determinants of flower color and antioxidant capacity in ornamental plants, few studies focus on the mechanisms of flavonoids that coordinate pigment accumulation with antioxidant capacity.

The key regulators of the flavonoid biosynthesis encompass structural genes and transcription factors, which have been extensively investigated in plant species (Dai and Hong, 2016). The biosynthesis of flavonoids is initiated by phenylalanine ammonia-lyase (*PAL*) and 4-coumarate-CoA ligase (*4CL*), with subsequent enzymatic modifications carried out by products of structural genes (Punyasiri et al., 2004). Moreover, the shikimate acyltransferase (*HCT*) consumes the flavonoid precursor p-coumaroyl-CoA to generate caffeoyl-CoA, consequently suppressing flavonoid biosynthesis (Hoffmann et al., 2004). Subsequently, numerous studies reveal that the differential expression of core-structural genes such as chalcone synthase (*CHS*), flavonol synthase (*FLS*), flavone 3’-hydroxylase (*F3’H*), flavone 3,5-hydroxylase (*F3’5’H*), dihydroflavonol 4-reductase (*DFR*), anthocyanidin synthase *(ANS*), and glucuronosyltransferase (*GT*) across different flower colors in *Rhododendron pulchrum*, *Tulipa fosteriana*, and *Grape Hyacinth* (Liu et al., 2019; Xia et al., 2022; Yuan et al., 2016). UDP-glucuronosyltransferase (*UGT*) such as *SoUGT1*, *PhUGT79B31*, and *NmF4’GT* regulate anthocyanin accumulation through 3-O-glucosylation and acylation, altering flower in *Syringa oblata*, *Petunia hybrida*, and *Nemophila menziesii* (Knoch et al., 2018; Okitsu et al., 2018; Petit et al., 2020; Wang et al., 2025). The MYB-bHLH-WD40 (MBW) complex precisely regulates the spatiotemporal expression of structural genes in the flavonoid biosynthesis pathway (Ramsay and Glover, 2005). The R3-MYB transcriptional repressor MYBx precisely modulates the spatial distribution of anthocyanin pigmentation in grape hyacinth (Zhang et al., 2020). Integrative multi-omics profiling reveals transcriptional networks governing flavonoid and anthocyanin biosynthesis, deciphering the molecular basis of anthocyanin-mediated pigmentation in *Malus*, *Morus alba*, *Clematis lanuginosa*, and *Cymbidium floribundum* (Ma et al., 2024; Qian et al., 2025; Xia et al., 2024; Zhao et al., 2024). Despite the central role of flavonoid biosynthesis in modulating floral pigmentation and antioxidant potential in ornamental plants, few studies focused on the metabolic flux partitioning of flavonoids for determinants of flower color and antioxidant capacity.

Lilac plants (*Syringa* spp.) play the important role in courtyard design and landscape architecture due to the plentiful varieties with elegant branches, colorful flowers, and fragrant flowers. The *Syringa* plants also exhibit medicinal and edible properties in barks, fruits, and flowers, attributed to a rich array of bioactive compounds including proteins, phenolics, iridoid glycosides, lignans, flavonoids, and gallic acid (Gąsecka et al., 2023; Jakubczyk et al., 2022; Tóth et al., 2016; Zhou et al., 2008; Zhu et al., 2021a). For example, delphinidin-3-O-rutinoside and cyanidin-3-O-rutinoside are the major anthocyanins responsible for petal pigmentation in *Syringa oblata* and *Syringa vulgaris* (Deeva et al., 2022; Ma et al., 2022). Some evidences by transcriptome, genomic, and metabolomic analysis presented structural genes (*CHS*, *DFR*, *ANS*, *F3H*, *F3’H*, *4CL*, and *PAL*) and transcription factors (WRKY and ERF) play key roles at different color transition stages, while the MBW complex is involved throughout the entire flower color formation process in *Syringa* species (Chen et al., 2022; Ma et al., 2022; Wang et al., 2022; Wang et al., 2025). Furthermore, the *Syringa* plants contains flavonoids like quercetin-3-O-rutinoside and kaempferol glucoside—polyphenolic compounds noted for their antioxidant properties (Jakubczyk et al., 2022). Bioactive phytochemicals from *Syringa* flowers and fruits regulate antioxidant activity by modulating ROS (El-Desouky and Gamal-Eldeen, 2009; Hanganu et al., 2021; Kurkin et al., 1989). The floral pigmentation pathways are linked to antioxidant flavonoid accumulation in *Syringa* plants. However, the current studies focused on the anthocyanidin-induced pigmentation or the flavonoid-driven antioxidant activity, respectively. Few works have concerned the dissection of the genetic and metabolic panorama, especially the metabolic flux partitioning in flavonoids that contribute to pigmentation and antioxidant abilities in *Syringa.*

In the study, the three cultivars of *S. vulgaris* with different petal colors are applied as experimental materials to investigate. By integrating phenotypic assays (total flavonoid and anthocyanin contents, radical scavenging capacity), flavonoid metabolomic profiling, and RNA sequencing, this study elucidated the physiological and molecular mechanisms governing corolla pigmentation and antioxidant capacity in three *S. vulgaris* varieties with distinct pigment phenotypes. Key regulatory genes and enzymes in flavonoid biosynthesis were identified, establishing a mechanistic framework for the metabolic allocation between anthocyanins and flavonoids. The study was promising to provide the theoretical insights into the regulatory networks governing floral pigmentation and antioxidant capacity, as well as the applicable potential for biobreeding for the desirable traits in lilac plants.

## Materials and methods

### Plant materials

**Figure 1:**
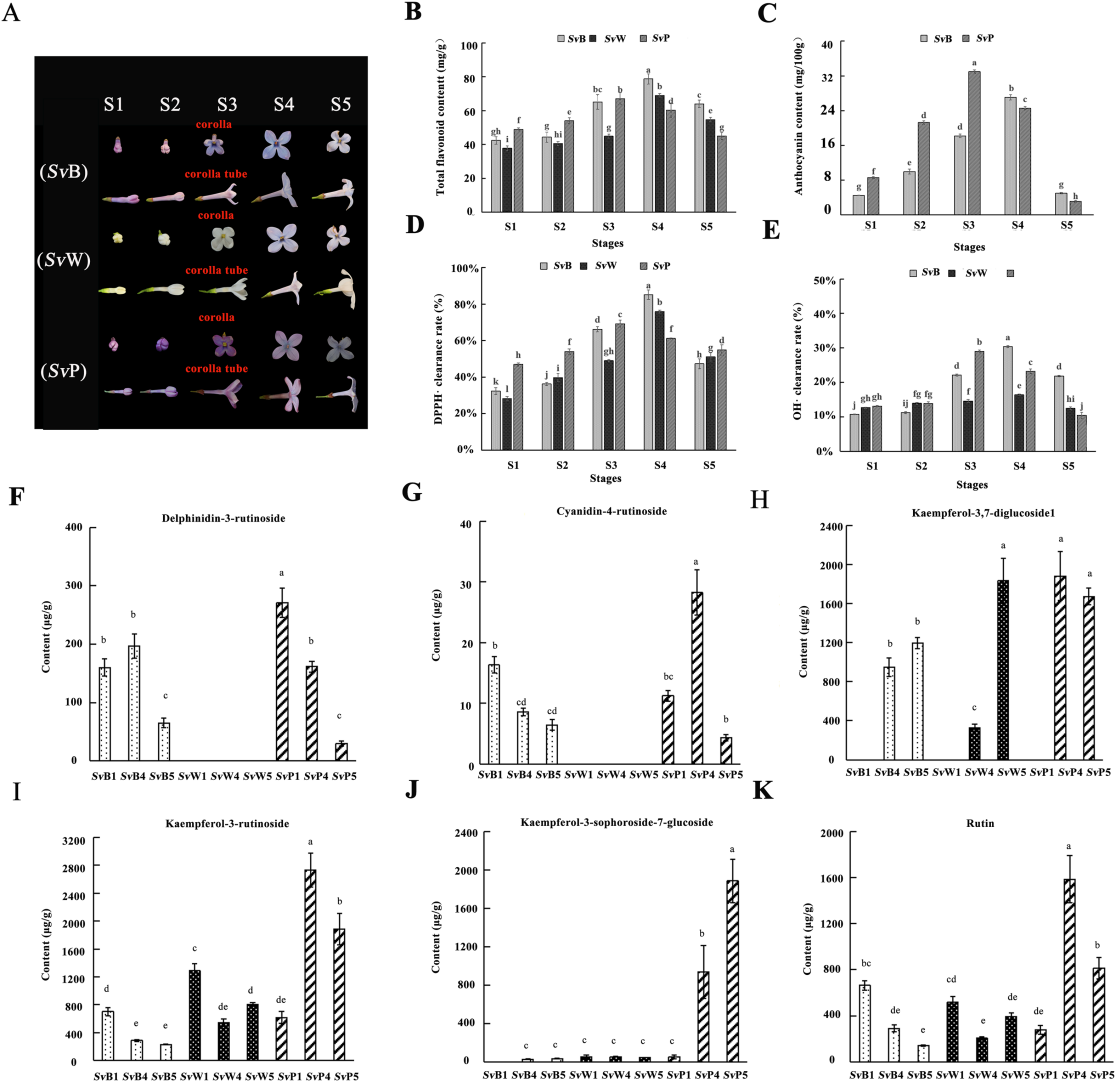
The comparative analysis of phenotypes, total flavonoids content, total anthocyanins content, 2,2-diphenyl-1-picrylhydrazyl radical (DPPH·) clearance, and hydroxyl radical (OH·) clearance rates, and metabolome profiling in three cultivars of *S. vulgaris.* **(A)** Phenotypes; **(B)** Total flavonoid (TF) content; **(C)** Total anthocyanin content; **(D)** 2,2-diphenyl-1-picrylhydrazyl radical (DPPH·) clearance rate; **(E)** hydroxyl radical (OH·) clearance rate; **(F)** Delphinidin-3-rutinoside; **(G)** Cyanidin-4-rutinoside; **(H)** Kaempferol-3,7-diglucoside; **(I)** Kaempferol-3-rutinoside; **(J)** Kaempferol-3-sophoroside-7-glucoside; **(K)** Rutin. S1-S5: The developmental stages, based on timely observation of the corolla open degree, referring to previous research, are divided into five stages, respectively, S1 (bud stage), S2 (flaw stage), S3 (semi-bloom stage), S4 (full-bloom stage) and S5 (late-bloom stage). *Sv*B: the blue *S. vulgaris* ‘Wedgewood Blue’; *Sv*W: the white *S. vulgaris* ‘Guifei III’; *Sv*P: the purple *S. vulgaris* ‘Guifei IV’. *Sv*B1, *Sv*W1, and *Sv*P1 represent *S. vulgaris* ‘Wedgewood Blue’, white *S. vulgaris* ‘Guifei III’, and purple *S. vulgaris* ‘Guifei IV’ at the bud stage (S1), respectively; *Sv*B4, *Sv*W4, and *Sv*P4 denote *S. vulgaris* ‘Wedgewood Blue’, white *S. vulgaris* ‘Guifei III’, and purple *S. vulgaris* ‘Guifei IV’ at the full-bloom stage (S4), respectively; *Sv*B5, *Sv*W5, and *Sv*P5 signify *S. vulgaris* ‘Wedgewood Blue’, white *S. vulgaris* ‘Guifei III’, and purple *S. vulgaris* ‘Guifei IV’ at the late-bloom stage (S5), respectively. Values followed by different letters indicate significant differences among the samples at p < 0.05.

The investigation employed the open-pollinated seed selection method for the blue cultivar *S. vulgaris* ‘Wedgewood Blue’ (*Sv*B) to cultivate two unique varieties: the white cultivar *S. vulgaris* ‘Guifei III’ (*Sv*W) and the purple cultivar *S. vulgaris* ‘Guifei IV’ (*Sv*P). In detailed observations of three *S. vulgaris* cultivars, their corollas exhibited pronounced color transitions across developmental stages (**Figure 1A**). However, the metabolic and molecular mechanisms underlying these color changes remain poorly understood, establishing *S. vulgaris* corollas as an exemplary model for investigating flavonoid and anthocyanidin flux partitioning in relation to floral pigmentation and antioxidant capacity.

In the study, the *Sv*B, as well as the offspring of the *Sv*W and *Sv*P, were cultivated on the germplasm nursery of Weifang Academy of Agricultural Sciences. Samples were collected from April 9^th^ to April 26^th^, 2022. Based on the flower-bloom stage of *S. vulgaris* in a previous study (Ji et al., 2012), we collected samples at five distinct developmental stages: bud stage (S1), bud break stage (S2), half-open stage (S3), full-bloom stage (S4), and late-bloom stage (S5). The corollas of inflorescence heads were collected from three different cultivars of *S. vulgaris*. Each cultivar was represented by three independent biological replicates. For each biological replicate, three technical replicates were performed during subsequent analyses to account for instrumental and procedural variability. Subsequently, the samples were immediately snap-frozen in liquid nitrogen and stored in a refrigerator maintained at -80 °Cfor subsequent analysis.

Initial profiling of phenotypic traits, total flavonoids, and anthocyanin content across five developmental stages revealed that S2 and S3 represent metabolically stable transitional stages, characterized by minimal flux variation and absence of regulatory inflection points, unlike the dynamic shifts observed in S1, S4, and S5 stages. Given their highly similar metabolic signatures and lack of distinct transcriptional or enzymatic transitions, inclusion samples of S2 and S3 stages in follow-up metabolomic and RNA sequencing analyses would not enhance resolution of key regulatory events governing flavonoid biosynthesis. Therefore, only samples from the S1, S4, and S5 stages were subjected to comprehensive omics profiling to maximize mechanistic insight into stage-specific metabolic flux partitioning.

### Determination of total flavonoid content

The 0.1 g sample was ground into a fine powder under liquid nitrogen conditions. The powder was extracted with 1 mL 60% ethanol using 500 W ultrasonic at 60 °C for 40 min, followed by centrifugation at 12,000 rpm at 25 °C for 12 minutes. The supernatant was measured using a microplate reader at 470 nm. A standard curve was constructed using the absorbance values of the rutin solutions at different concentrations, from which a regression equation was derived. The concentrations were calculated based on the regression equation. Finally, the formula for total flavonoid content was according to reported methods (Han et al., 2023) The total flavonoid content was determined as follows [V_Extraction_ w volume of added extraction, W was sample weight]:

Total flavonoid content (mg/g) = X × V_Extraction_/W

### Determination of total anthocyanin content

The 0.1 g sample was pulverized under liquid nitrogen. The powder was extracted using 1 mL of extraction solution, maintained at a temperature of 75 °C for a period of 40 minutes. Afterwards, the homogenate was centrifuged at 12,000 rpm for 10 minutes. The supernatant was adjusted to a final volume of 1 mL, and absorbance of the supernatant was measured using a microplate reader at 530 nm and 700 nm, respectively. The total anthocyanin content was determined as follows [A^sample^ and A^blank^ was the absorbance of sample and blank tube, respectively. D was dilution multiple, W was sample weight]:

ΔA =(A^sample530^ - A^sample700^) - (A^blank530^ - A^blank700^)

Total anthocyanin content (mg/g) = 0.0334 × ΔA/W × D

### Determination of 2,2-Diphenyl-1-picrylhydrazyl radical clearance rate

The 0.1 g powder of sample that was ground under liquid nitrogen was mixed with 1 mL of ethanol and incubated at 40 °C for a duration of 30 minutes. Subsequently, the homogenate was centrifuged at 4000 rpm for 10 min at 4 °C. The supernatant was added to other reagents according to the instructions, vortexed, and incubated at room temperature for 30 minutes. Afterward, the absorbance of supernatant was measured at 515 nm using a microplate reader. The clearance rate of DPPH· was determined as follows [A^sample^, A^control^, and A^blank^ were absorbancea of sample, control, and blank tube, respectively]:

DPPH· clearance rate (%) = [(A^blank^- (A^sample^ - A^control^))/A^blank^] × 100%

### Determination of hydroxyl radical (OH·) clearance rate

The 0.1 g sample was pulverized in liquid nitrogen and homogenized with1 mL extract solution. The homogenate was centrifuged at 12,000 rpm for 10 minutes at a temperature of 4 °C. The resulting supernatant was added to the reagents in accordance with the instructions and incubated at 37 °C for one hour, followed by centrifugation at 12,000 rpm for 10 minutes at room temperature. The absorbance of supernatant was measured at 536 nm using a microplate reader. The clearance rate of OH· was determined as follows [A^sample^, A^control^, and A^blank^ were absorbances of sample, control, and blank tube, respectively]:

OH· clearance rate (%) = [(A^sample^ - A^control^)/(A^blank^ - A^control^)] × 100%

### Flavonoids and anthocyanins metabolomic profiling

At the beginning, 0.5 g of the samples that thawed after undergoing liquid nitrogen grinding were placed within a 15 mL brown glass, and then 1 mg of peony 3-glucoside and 20 mg of myricetin-3-O-galactoside were added and thoroughly mixed with the samples. The resultant mixture was adjusted to a total volume of 4 mL with a methanol/formic acid solution (9:1, v/v) and stored in a refrigerator set at -4 °C for 12 hours. The supernatant was collected after centrifugation (2,000 rpm, 10 min), transferred to an amber vial, and subsequently dried under a gentle stream of nitrogen gas. To the remaining residue, 200 μL of methanol/formic acid solution (9:1, v/v) was added. The solution was thereafter filtered through a 0.22 μm nylon membrane to eliminate impurities for further analysis. The secondary metabolites of flavonoids and anthocyanins were quantified utilizing an ultra-performance liquid chromatography-quadrupole time-of-flight tandem mass spectrometer (UPLC-Q-TOF-MS).

### RNA sequencing data analysis

Total RNA was isolated from the three cultivars at three critical developmental stages (S1, S3, and S5) utilizing the RNAprep pure plant kit (Tiangen, Beijing, China), and the purity and concentration was evaluated using a Nanodrop 2000C spectrophotometer (Thermo Scientific, Vermont, USA) and 1% (w/v) agarose gel electrophoresis. The construction of the cDNA libraries and the subsequent transcriptome sequencing were carried out by Biomarker Technologies Co., Ltd. (Beijing, China). For each sample, the mRNA was enriched using oligo (dT)-rich magnetic beads, then fragmented and used to synthesize first-strand cDNA with random primers. The second strand of cDNA was synthesized using RNase H and DNA polymerase I. Afterward, the cDNA underwent repair, poly(A) tailing, and adapter ligation. The cDNA fragments of the appropriate size were separated via agarose gel electrophoresis and amplified through PCR to generate the cDNA libraries for RNA-sequencing. The quality of the sample libraries was determined by an Agilent 2100 Bioanalyzer and Qubit 2.0. Utilizing the Sequencing By Synthesis (SBS) technology, the Illumina high-throughput sequencing platform was employed to sequence the cDNA libraries, yielding extensive and high-quality raw data.

### Functional gene annotation and differential expression analysis

The sequencing data collected from the samples was trimmed using Trimmomatic-0.39 with parameter AVGQUAL 30. HISAT2 was subsequently employed to align the clean data with the reference genome of *S. oblata* (NGDC, https://ngdc.cncb.ac.cn/), thereby generating mapped data. The normalization process used the number of mapped reads and their transcript length, calculated using the FPKM metric (Trapnell et al., 2010). This FPKM metric serves as an indicator of the transcript or gene expression level, effectively illustrating the gene expression level. Genes were considered significantly differentially expressed if they exhibited a log2 FC ≥ 2 and a q-value < 0.05. The sequences of these differentially expressed genes (DEGs) were aligned to various databases, including Clusters of Orthologous Groups (COG), Gene Ontology (GO), Kyoto Encyclopedia of Genes and Genomes (KEGG), Eukaryotic Orthologous Groups (KOG), Protein Family (Pfam), and the manually annotated and BLAST-reviewed protein sequence databases, including Swiss-Prot and Non-redundant (NR), were utilized. Through this alignment procedure, relevant annotation data were acquired. We performed Pearson correlation analysis to assess the relationship between the two variables.

### Quantitative real-time PCR

Ten DEGs were chosen at random, and specific primers were designed using online software (https://www.genscript.com) and produced by Jin Weizhi Biotechnology (Beijing) Co., Ltd. β-actin (GenBank accession No. AF545569.1) was selected as the reference gene, and a specific primer pair was designed and validated for quantitative real-time PCR (qRT-PCR) applications. The qRT-PCR was carried out following the fluorescence quantification guidelines (Jędrzejuk et al., 2016). Relative expression levels were computed using the 2^- △△CT^ method.

### Statistical analysis

Statistical analysis was conducted using Office 2020 (Microsoft, Redmond, WA, USA), IBM SPSS Statistics 2019 (SPSS Inc., Chicago, IL, USA), and Origin 2019 (Origin Lab, Northampton, MA, USA). Statistical significance was assessed via an independent-sample *t*-test or one-way ANOVA with Duncan’s multiple comparisons test. Pearson correlation coefficient was employed for the correlation analysis. Each group’s biological experiment was repeated three times, with five technical replicates, and results were presented as mean ± standard deviation.

## Results

### Total flavonoid and anthocyanin content analysis

Analysis of total flavonoid content was carried out in corollas of the three cultivars at S1, S2, S3, S4, and S5 stages (**Figure 1A**). Our analysis delineated a distinct three-cultivar trajectory of flavonoid dynamics throughout flower development. The flavonoids accumulated to peak levels in early stage flowers, then, a progressive but marked reduction occurred in parallel with the sequential stages of floral tissue differentiation and senescence. The content of total anthocyanin in *Sv*B and *Sv*P cultivars was consistent with the dynamics of total flavonoid content, with the peak content being reached at the S3 and S4 stages, respectively (**Figure 1B**). The total anthocyanin content was below the limit detected in *Sv*W cultivars (**Figure 1C**). The *Sv*P exhibited maximum content of total flavonoid at the S3 stage, while the *Sv*B and *Sv*W peaked at the S4 stage. Across all *S. vulgaris* cultivars, we pinpointed the S1, S4, and S5 as pivotal developmental stages characterized by substantial and reproducible shifts in flavonoid metabolism.

Flavonoid accumulation exhibited cultivar-specific dynamics across flower developmental stages, with distinct temporal patterns established during maturation. The *Sv*W cultivar consistently exhibited lower total flavonoid content than *Sv*B across all developmental stages, while the *Sv*P cultivar maintained a higher total flavonoid content than the *Sv*B cultivar during the S1 and S2 stages, but surpassed neither *Sv*B nor *Sv*W at the S4 and S5 stages, where its content declined markedly. Concurrently, anthocyanin concent in *Sv*W remained below the limit (LOD) of detection throughout development. These results revealed that total flavonoid and anthocyanin accumulation exhibited pronounced spatiotemporal variation across developmental stages and cultivars, collectively shaping the distinct pigmentation profiles that differentiated the three genotypes.

### DPPH· and OH· scavenging rates analysis

The measurements of scavenging rates of OH· and DPPH· were conducted in three cultivars of *S. vulgaris* at S1–S5 stages. The scavenging rate of DPPH· presented a peak at the S4 stage in *Sv*B, with values ranging from 32.38% to 85.03% (**Figure 1D**). The scavenging rates of DPPH· correlated closely with total flavonoid content across all developmental stages: *Sv*W ranged from 28.25% to 75.85%, while *Sv*P ranged from 46.93% to 69.21%. The *Sv*P exhibited significantly higher scavenging activity than both *Sv*B and *Sv*W at all stages except S4 stage, where *Sv*B surpassed it. Conversely, *Sv*W demonstrated significantly greater activity than *Sv*B at S2 and S5 stages, whereas *Sv*B showed superior activity at S1, S3, and S4 stages. The scavenging rates of OH· ranged from 10.75–30.34% for *Sv*B, 12.45–16.39% for *Sv*W, and 10.47–29.00% for cultivar *Sv*P throughout the developmental stages (**Figure 1E**). During early developmental stages, *Sv*B showed weaker OH· scavenging activity compared to *Sv*W (before S3) and *Sv*P (before S4). A striking shift occurred after the S3 stage, where *Sv*B’s OH· scavenging capacity increased dramatically and exceeded that of the other two cultivars. Meanwhile, *Sv*W displayed relatively consistent OH· scavenging rates across all stages, with significantly lower activity than *Sv*P at S3 and S4 but higher activity at S5.

Across the three cultivars, radical scavenging activity exhibited a consistent biphasic trend throughout flower development, rising initially before declining progressively. Stages S1, S3, and S4 were identified as critical inflection points for both DPPH· and OH· scavenging rates. This comparative analysis suggested that differential radical scavenging capacities among the cultivars. The scavenging rates of DPPH· and OH· were consistent with the total flavonoid content, the *Sv*B and *Sv*P cultivars exhibiting significant DPPH· and OH· scavenging capacities at specific developmental stages, respectively. These results implied a correlation between flavonoid metabolism and antioxidant activity in the three cultivars of *S. vulgaris*.

### The correlation analysis between the flavonoid content and radical scavenging

Correlation analysis demonstrated that total flavonoid content exhibited significant positive relationships with the scavenging rates of DPPH· and OH· throughout development in all three *S. vulgaris* cultivars (**Supplementary Table 1**). Significant correlations were found between the total flavonoid content and the scavenging rates of OH· and DPPH· in the *S. vulgaris.* Similarly, total anthocyanin content was positively correlated with both radical scavenging metrics, reinforcing their functional synergy in antioxidant defense. The consistent pattern across cultivars suggested that anthocyanins are not merely pigments but integral components of the antioxidant network in *S. vulgaris.*

### Metabolome analysis

Anthocyanin and flavonoid metabolites were subjected to targeted profiling using UPLC-Q-TOF-MS. Two anthocyanin metabolites were identified in the *Sv*B and *Sv*P cultivar: delphinidin-3-rutinoside and cyanidin-4-rutinoside, neither of which were detected above the LOD in the *Sv*W cultivar (**Supplementary Table 2**). In the two cultivars, delphinidin-3-rutinoside dominated, accounting for over 87.3% of the total anthocyanin content, underscoring its role as the principal chromophore driving coloration in these genotypes. The *Sv*B cultivar exhibited a biphasic accumulation pattern of delphinidin-3-rutinoside during development, peaking at 197.15 μg/g in the S4 stage, followed by a decline (**Figure 1F**). Conversely, delphinidin-3-rutinoside content in *Sv*P decreased progressively across the three developmental stages, peaking at 271.16 μg/g at S1 Stage. Before the S4 stage, the *Sv*P cultivar displayed significantly higher content of delphinidin-3-rutinoside compared to the *Sv*B cultivar. However, *Sv*B experienced a marked increase in delphinidin-3-rutinoside, surpassing that of *Sv*P at the S5 stage (**Figure 1F**). The biosynthetic trajectory of cyanidin-4-rutinoside differed markedly between cultivars: *Sv*B displayed a monotonic decline, while *Sv*P followed a biphasic pattern that accumulated to a transient maximum before S4, then declining (**Figure 1G**). Although *Sv*P generally contained lower content than *Sv*B at most stages, a statistically significant elevation was observed at S4 stage indicating a cultivar-and stage-specific regulatory switch in anthocyanin metabolism.

We identified four flavonoid metabolites (kaempferol-3,7-diglucoside, kaempferol-3-sophorose-7-glucose, kaempferol-3-rutinoside, and rutin) in the three cultivars of *S. vulgaris* (**Supplementary Table 2**). In the *Sv*B and *Sv*W: kaempferol-3,7-diglucoside became the dominant constituent (>61.7% of total flavonoid metabolites) at S4 and S5 stages, while kaempferol-3-rutinoside and rutin were the most abundant at S1 stage, implying stage-specific regulation of glycosyltransferase activity. In *Sv*P, flavonoid metabolites remained evenly distributed throughout development, with relative proportions of all major glycosides fluctuating, indicating a lack of dominant metabolic flux toward any single pathway branch. The kaempferol-3,7-diglucoside was undetectable in all cultivars at the S1 stage (**Figure 1I**). In *Sv*B and *Sv*W, its content increased progressively across developmental stages, whereas *Sv*P exhibited a biphasic pattern that increased to a peak followed by a decrease. At the S4 and S5 stages, the *Sv*B cultivar contained lower content of kaempferol-3,7-diglucoside than the *Sv*P, while the *Sv*B was significantly higher than the SvW cultivar at the S4 stage. No significant difference existed in the content of kaempferol-3,7-diglucoside between *Sv*W and *Sv*P at S5 stage. In contrast, kaempferol-3-rutinoside content in *Sv*B cultivar decreased monotonically throughout development. *Sv*W displayed a biphasic trend, with an initial decrease followed by a marked increase, while *Sv*P followed the opposite pattern: an initial increase followed by a sustained decrease (**Figure 1I**). Conversely, the kaempferol-3-rutinoside content of *Sv*P increased initially and then decreased. The *Sv*W was higher in content of kaempferol-3-rutinoside than in *Sv*B but lower than in *Sv*P at the S4 and S5 stages. The kaempferol-3-sophorose-7-glucose exhibited a sustained, stage-dependent accumulation in *Sv*P, increasing progressively from S1 to S5, while levels in *Sv*B and *Sv*W remained stably low with no significant fluctuation across development (**Figure 1J**). By the S4 and S5 stages, *Sv*P accumulated significantly higher content than both *Sv*B and *Sv*W. The content of rutin showed similar results to kaempferol-3-rutinoside in three *S. vulgaris* cultivars (**Figure 1K**). These different flavonoid metabolite accumulation trajectories across cultivars and developmental stages, which suggested differential regulation of flavonoid biosynthetic pathways in *S. vulgaris*.

### Integrated physiological and metabolomics analysis

A correlation analysis was performed between flavonoid and anthocyanin metabolites and the scavenging rates of DPPH· and OH·, as well as the total flavonoid content in three cultivars of *S. vulgaris.* The lack of significant correlation between total flavonoid content and scavenging rates of DPPH· and OH· in *Sv*B, SvW, and *Sv*P cultivars implied that flavonoid metabolites do not serve as the principal direct antioxidants in these tissues. Nevertheless, cyanidin-4-rutinoside demonstrated a significant and positive correlation with the total anthocyanin content. These findings indicated that cyanidin-4-rutinoside serves as a dominant anthocyanin component across all three cultivars of *S. vulgaris*, playing a key role in modulating floral pigmentation.

### Functional annotation and classification of unigenes

The results demonstrated that a total of 171.29 GB of clean data was acquired in this experiment, with an average of 5.76 GB per sample. Notably, reads containing more than 10% of N were not detected in the data. The Q30 base percentage reached 91.44% and above, and the GC content was between 43.22% and 44.21%. The high-quality clean reads of each sample were mapped to the reference genome, *S. oblata*, and the mapping efficiency ranged from 73.96% to 86.83%, which indicated a close genetic relationship between *S. oblata* and *S. vulgaris* (**Supplementary Tables 3**, **4**). As above, and the GC was between 43.22% and 44.21%. Sequence alignment demonstrated that more than 80% of the conserved genes shared between the two species exhibited high nucleotide sequence similarity, thereby corroborating their close phylogenetic relationship. Additionally, annotations were identified for 47,981 unigenes in the NR database, 23,930 in KOG, 37,782 in GO, 30,845 in SwissProt, and 35,426 in Pfam (**Supplementary Table 5**). These results fully demonstrated the reliability of the test quality and can be used for subsequent analysis.

### Differently expressed genes analysis by RNA-seq

Initially, we conducted an analysis of the differentially expressed genes (DEGs) in accordance with the screening criteria of Log_2_ fold-change (Log_2_FC) > 2 and false discovery rate (q) < 0.05. For deep analysis, DEGs were divided into gene sets for assay. The specific details of the gene-set-analysis groups were presented (**Supplementary Tables 6**, **7**). Our analysis revealed that 899, 1524, and 3695 DEGs were identified in the B-d, W-d, and P-d groups, respectively, whereas 1742, 1563, and 1958 DEGs were detected in the BW-d, BP-d, and WP-d groups, respectively. The DEGs exhibited alterations in expression levels across the three stages and cultivars.

KEGG pathway annotation was carried out in the different gene-set-analysis groups (**Supplementary Table 8**). Across the *Sv*B, *Sv*W, and *Sv*P developmental stages, DEGs were significantly enriched in a variety of biological pathways. In *Sv*B (B-a, B-b, B-c groups), DEGs enriched included plant-pathogen interaction, plant hormone signal transduction, starch and sucrose metabolism, MAPK signaling pathway-plant, carbon metabolism, phenylpropanoid biosynthesis, biosynthesis of amino acids, and ribosome (**Supplementary Table 8**; **Supplementary Figure 1A**). In *Sv*W (W-a, W-b, W-c groups), pathways such as plant-pathogen interaction, plant hormone signal transduction, starch and sucrose metabolism, mannose type O-glycan biosynthesis, glycerophospholipid metabolism, biosynthesis of unsaturated fatty acids, MAPK signaling pathway-plant, and phenylpropanoid biosynthesis were enriched (**Supplementary Table 8**; **Supplementary Figure 1B**). In *Sv*P (P-a, P-b, P-c groups), enrichments covered plant-pathogen interaction, plant hormone signal transduction, amino acid biosynthesis, carbon metabolism, MAPK signaling pathway-plant, endoplasmic reticulum protein processing, pentose and glucuronate interconversions, starch and sucrose metabolism, and phenylpropanoid biosynthesis (**Supplementary Table 8**; **Supplementary Figure 1C**).

In DEGs between *Sv*B and *Sv*W cultivars, a large number of DEGs were enriched in plant-pathogen interaction, plant hormone signaling, starch and sucrose metabolism, MAPK signaling pathway-plant, carbon metabolism, biosynthesis of amino acids, protein processing in endoplasmic reticulum, and phenylpropanoid biosynthesis (**Supplementary Table 8**; **Supplementary Figure 2A**). In DEGs between *Sv*B and *Sv*P cultivars, DEGs were encompassed in ribosomes, plant-pathogen interaction, plant hormone signaling, starch and sucrose metabolism, MAPK signaling pathway-plant, phenylpropanoid biosynthesis, carbon metabolism, and biosynthesis of amino acids (**Supplementary Table 8**; **Supplementary Figure 2B**). Between *Sv*B and *Sv*P cultivars, most DEGs were predominantly represented in plant-pathogen interaction, plant hormone signal transduction, MAPK signaling pathway-plant, protein processing in endoplasmic reticulum, starch and sucrose metabolism, pentose and glucuronate interconversion, and endocytosis (**Supplementary Table 8**; **Supplementary Figure 2C**).

**Figure 2:**
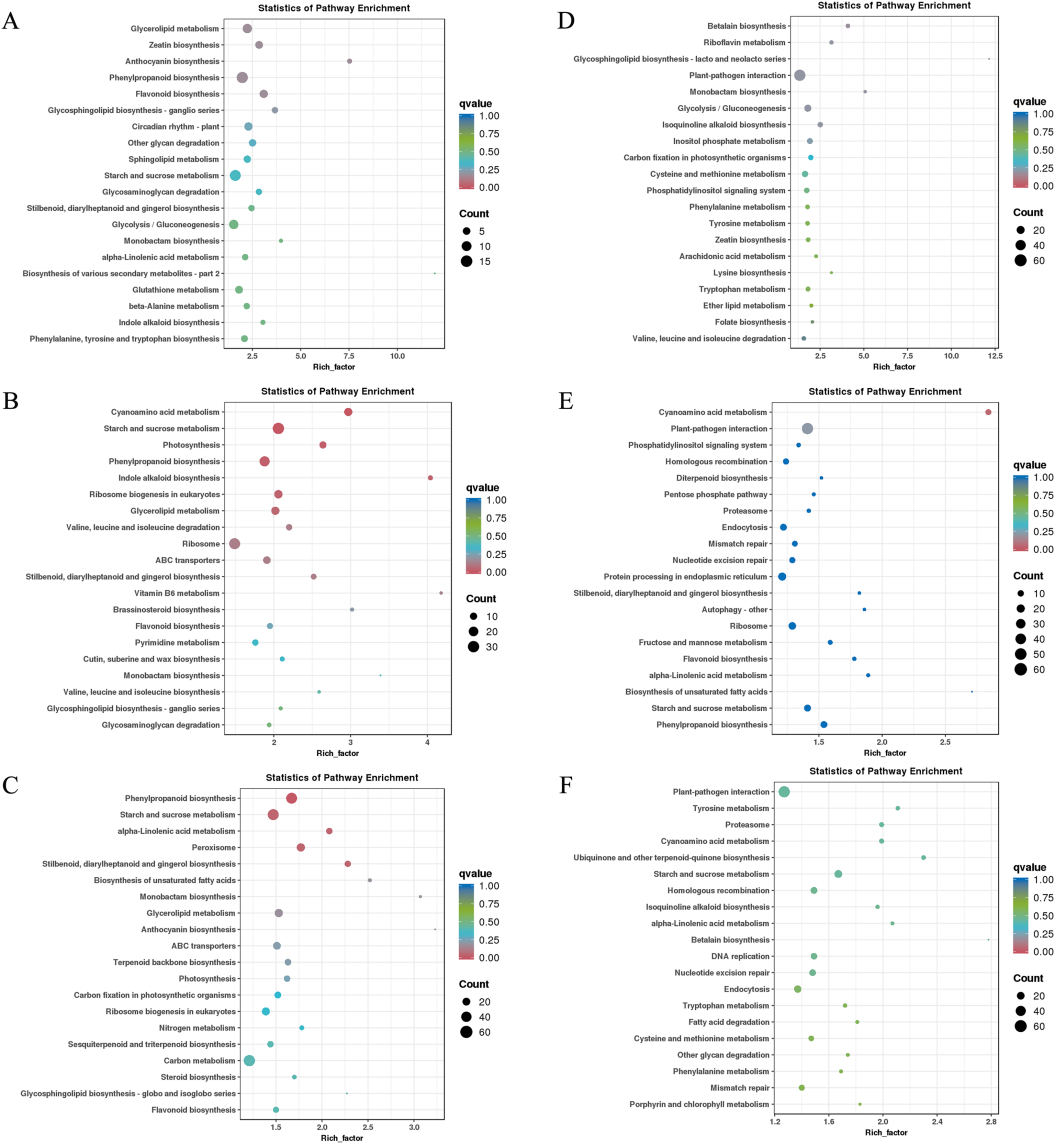
KEGG enrichment of DEGs in gene-set-analysis groups. **(A)** Dotplot of DEGs in the B-d group; **(B)** Dotplot of DEGs in the W-d group; **(C)** Dotplot of DEGs in the P-d group; **(D)** Dotplot of DEGs in the BW-d group; **(E)** Dotplot of DEGs in the BP-d group; **(F)** Dotplot of DEGs in the WP-d group. The DEGs between *Sv*B1 and *Sv*B4, those between *Sv*B4 and *Sv*B5, and those between *Sv*B1 and *Sv*B5 were respectively designated as the gene-set-analysis groups B-a, B-b, and B-c (**Supplementary Table 6**). The same method was used for both *Sv*W and *Sv*P varieties. The DEGs between *Sv*B1 and *Sv*W1, the DEGs between *Sv*B4 and *Sv*W4, and those between *Sv*B5 and *Sv*W5 were respectively designated as the gene-set-analysis groups BW-a, BW-b, and BW-c (**Supplementary Table 6**). The same grouping method was used for the differential genes among other varieties. The intersection sets of B-a and B-b, W-a and W-b, and P-a and P-b were named as B-d, W-d, and P-d gene-set-analysis groups, respectively (**Supplementary Table 6**). These DEGs in these groups consistently changed their expression levels at the three stages. The intersection set of BW-a, BW-b, and BW-c was named the BW-d gene-set-analysis group. The intersection set of BP-a, BP-b, and BP-c was named the BP-d gene-set-analysis group. The intersection set of WP-a, WP-b, and WP-c was named the WP-d gene-set-analysis group. Genes of BW-d, BP-d, and WP-d gene-set-analysis groups were consistently kept as differentially expressed between the three cultivars of *S. vulgaris* when they were compared to each other. What’s more, the total gene numbers, up-expressed gene numbers, and down-expressed gene numbers of each gene-set-analysis group were shown in **Supplementary Table 6** and **7**.

Across the six gene-set-analysis groups (B-d, W-d, P-d, BW-d, BP-d, WP-d), the DEGs were significantly enriched in canonical pathways including plant–pathogen interaction, plant hormone signal transduction, starch and sucrose metabolism, MAPK signaling, and ribosomal function (**Supplementary Table 8**; **Figure 2**). Crucially, flavonoid biosynthesis, anthocyanin biosynthesis, and flavone and flavonol biosynthesis exhibited consistent and significant enrichment in every group, underscoring their non-redundant role in cultivar-specific metabolic reprogramming. Notably, the B-d and P-d groups displayed the most pronounced enrichment in anthocyanin biosynthesis, while the W-d, BW-d, BP-d, and WP-d groups retained substantial activation across the broader flavonoid network. Additional pathways of glutathione metabolism, α-linolenic acid metabolism, and zeatin biosynthesis further delineated cultivar-specific metabolic priorities. Collectively, these data revealed that anthocyanin biosynthesis was not merely a co-activated process but emerged as a defining molecular signature that discriminates between *S. vulgaris* cultivars, likely underpinning divergent floral pigmentation and stress-responsive flavonoid partitioning.

### DEGs analysis in flavonoid-related metabolism

**Figure 3:**
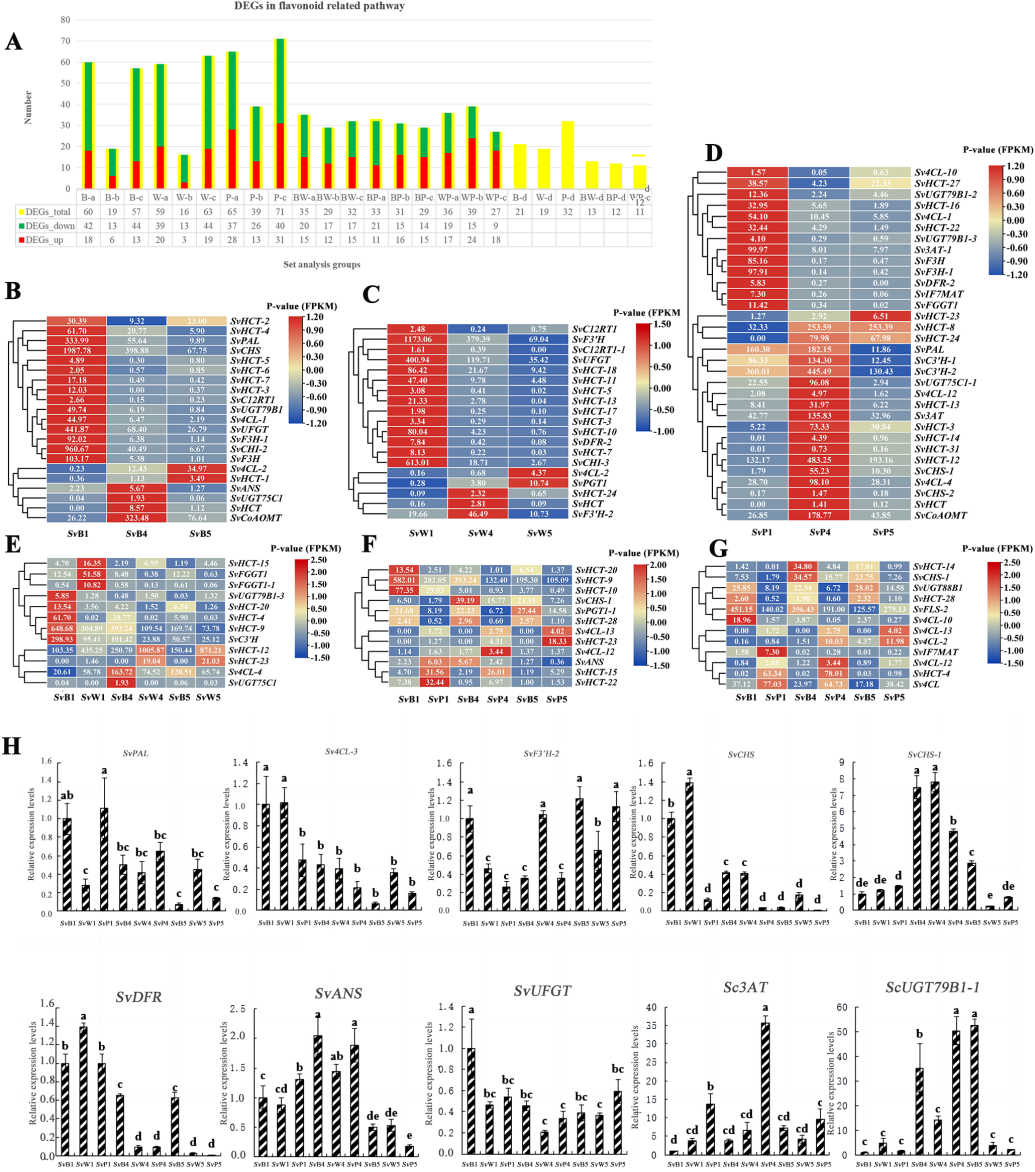
Analysis of candidate differentially expressed genes (DEGs) associated with flavonoid metabolism in three cultivars of *S. vulgaris.* **(A)** DEGs related to flavonoids in gene-set-analysis groups; **(B)** DEGs present at developmental stages in *S. vulgaris* ‘Wedgewood Blue’; **(C)** DEGs present at all developmental stages in *S. vulgaris* ‘Guifei III’; **(D)** DEGs present at developmental stages in *S. vulgaris* ‘Guifei IV’; **(E)** DEGs between *S. vulgaris* ‘Wedgewood Blue’ and *S. vulgaris* ‘Guifei III’ present at developmental stages; **(F)** DEGs between *S vulgaris* ‘Wedgewood Blue’ and *S. vulgaris* ‘Guifei IV’ present at developmental stages; **(G)** DEGs between *S. vulgaris* ‘Guifei III’ and *S. vulgaris* ‘Guifei IV’ present at developmental stages; **(H)**: Analysis of DEGs by qRT-PCR. The white numbers on the heatmap in the figure represent the fragments per kilobase of transcript per million mapped reads (FPKMs) of the DEGs. Values followed by different letters indicate significant differences among the samples at p < 0.05.

We mined a large number of DEGs related to the flavonoid metabolism in different gene-set-analysis groups (**Figure 3A**; **Supplementary Table 10**). In this study, we discovered that *HCTs* constituted the most significant component of the DEGs, with 34 *HCT*s identified across various gene-set-analysis groups (**Supplementary Figure 4B**). We found that down-regulated genes outnumbered up-regulated genes across developmental stages in three *S. vulgaris* cultivars. Expression levels varied significantly among cultivars and stages, with genes up-regulated or down-regulated depending on these factors. These results indicated that these genes regulated a complex flavonoid mechanism in *S. vulgaris*, contributing to differences between cultivars and their developmental stages. Ten genes were randomly selected for validation via qRT-PCR, including *SvPAL*, *Sv4CL-3*, *SvCHS*, *SvCHS-1*, *SvF3’H*, *SvDFR*, *SvANS*, *SvUFGT*, *SvUGT79B1-1*, and glucoside malonyltransferase gene (*Sv3AT*) (**Figure 3H**). The expression profiles of these genes, as quantified by qRT-PCR, exhibited strong concordance with the transcript levels derived from RNA-seq data, thereby validating the reliability of the RNA-sequencing data for research.

We extracted flavonoid-related DEGs from the B-d, W-d, P-d, BW-d, BP-d, and WP-d gene-set-analysis groups, with 21, 19, 32, 13, 12, and 12 DEGs, respectively (**Figure 3A**). In the B-d group, most DEGs were significantly down-regulated (**Figure 3B**). However, *SvHCT*, *SvANS*, and *SvUFT75C1* exhibited an initial up-regulation followed by down-regulation, whereas *Sv4CL-2* and S*vHCT-1* remained up-regulated throughout development. Similar expression patterns were observed in the W-d group (**Figure 3C**). In the P-d group, half of the flavonoid-related DEGs peaked at the S4 stage, while the remaining half showed sustained down-regulation (**Figure 3D**). These results indicated that multiple flavonoid biosynthesis genes were dynamically regulated during developmental transitions across the three *S. vulgaris* cultivars, with *Sv*P appearing to regulate a distinct subset of these genes under special transcriptional regulation. Moreover, one *4CL*, one coumaroylquinate 3’-Hydroxylase gene (*C3’H*), six *HCT*s, and four *GT*s in BW-d, had significantly different expression levels between the *Sv*B and *Sv*W *S. vulgaris* cultivars at the three stages (**Figure 3E**). Two *4CL*s, five *HCT*s, one *CHS*, one phlorizin synthase gene (*PGT*), and one *ANS* always existed in the DEGs between the *Sv*B and *Sv*P cultivars (**Figure 3F**). There were continual expression differences in five *4CL*s, three *HCT*s, one *FLS*, one *CHS*, and two *AT*/*GT*s during developmental stages of the *Sv*W and *Sv*P cultivars (**Figure 3G**). The results showed that different cultivars of *S. vulgaris* have different mechanisms for flavonoids, and *4CL*s and *HCT*s may play an important role in the mechanisms.

**Figure 4:**
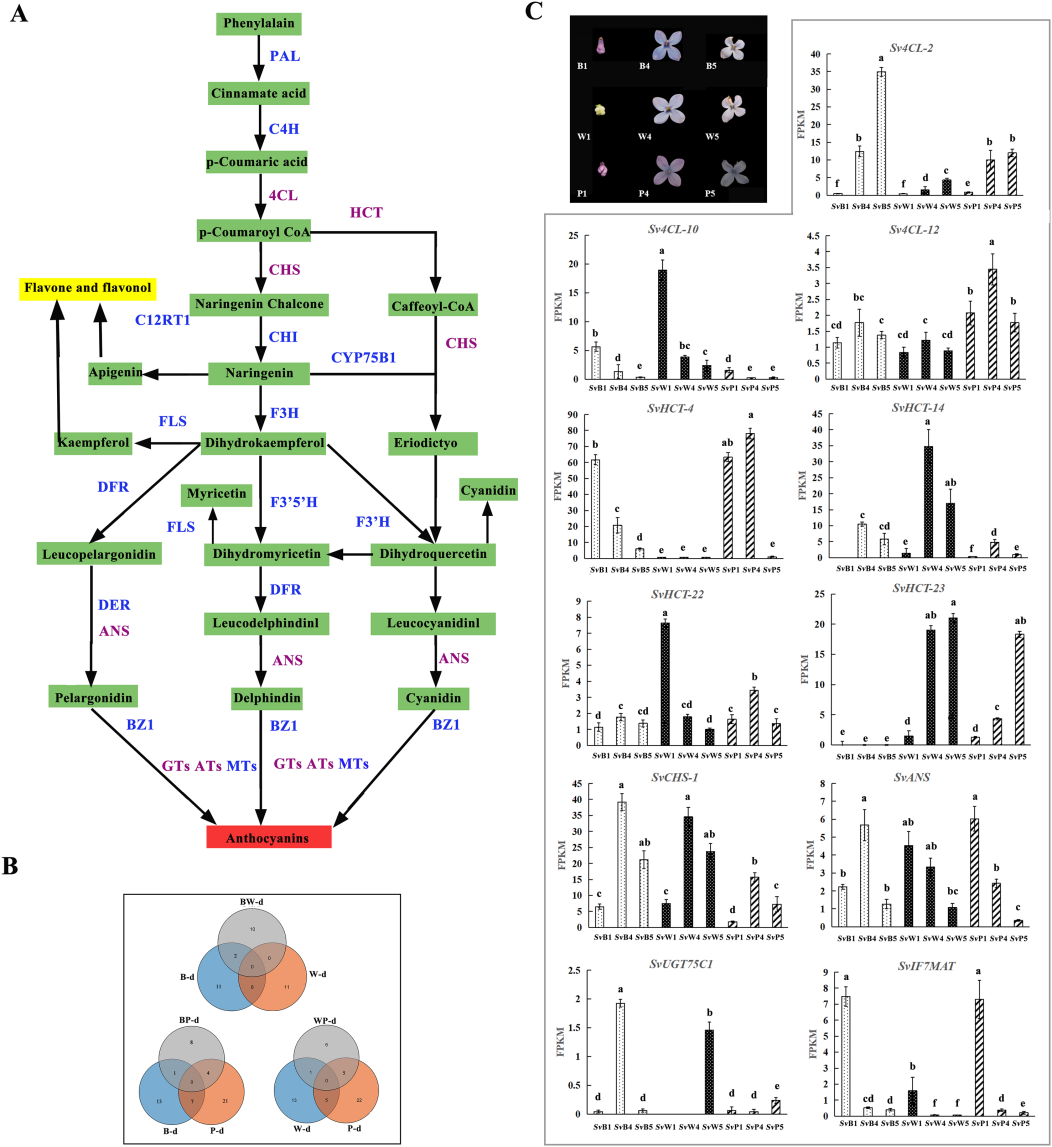
DEGs related to flavonoids co-existing in genotypes and developmental stages in the three cultivars of *S. vulgaris.* **(A)** DEGs analysis in flavonoid metabolism; **(B)** Venn diagram depicting differentially expressed genes (DEGs) across six set-analysis groups; **(C)** FPKMs of flavonoid-related DEGs co-existing in cultivar and developmental stages of *S. vulgaris.* Values followed by different letters indicate significant differences among the samples at p < 0.05. The designation of the red gene reveals the candidate DEGs.

A total of 11 flavonoid-related DEGs were identified across the B-d, W-d, P-d, BW-d, BP-d, and WP-d groups, revealing cultivar and development stage-specific transcript dynamics in *S. vulgaris* (**Figure 4**). In *Sv*B, *Sv4CL-2*, *SvHCT-4*, *SvANS*, and *SvUGT75C1* exhibited varying expression at three stages (**Figure 4B**). Notably, *Sv4CL-2* expression differed significantly between *Sv*W and *Sv*P, while *SvHCT-4* was consistently upregulated in *Sv*B compared to *Sv*W across the three stages. The *SvANS* and *SvUGT75C1* in *Sv*B showed significantly higher expression than in *Sv*P and *Sv*W, respectively. The *Sv4CL-10* in *Sv*B varied across developmental stages and was markedly suppressed relative to *Sv*W. In *Sv*P, the *Sv4CL-12* and *SvHCT-14* displayed stage-dependent expression, with *Sv4CL-12* significantly elevated compared to both *Sv*W and *Sv*B, whereas *SvHCT-14* was repressed relative to *Sv*W. The *SvHCT-22* and *SvHCT-23* in *Sv*P also exhibited developmental regulation and were significantly divergent from *Sv*W and *Sv*B, respectively. The *SvCHS-1* expression in *Sv*P varied across stages and was consistently lower than in both *Sv*W and *Sv*B. The isoflavone 7-O-glucoside-6’-O-malonyltransferase gene (*SvIF7MAT*) in *Sv*P followed a declining trend during development but remained consistently higher than in *Sv*W across all stages. Collectively, these spatiotemporal expression patterns implicate these DEGs as key regulators underlying the cultivar-specific divergence in flavonoid metabolism during flower development in the *S. vulgaris.*

### DEGs analysis in antioxidant-related pathways

**Figure 5:**
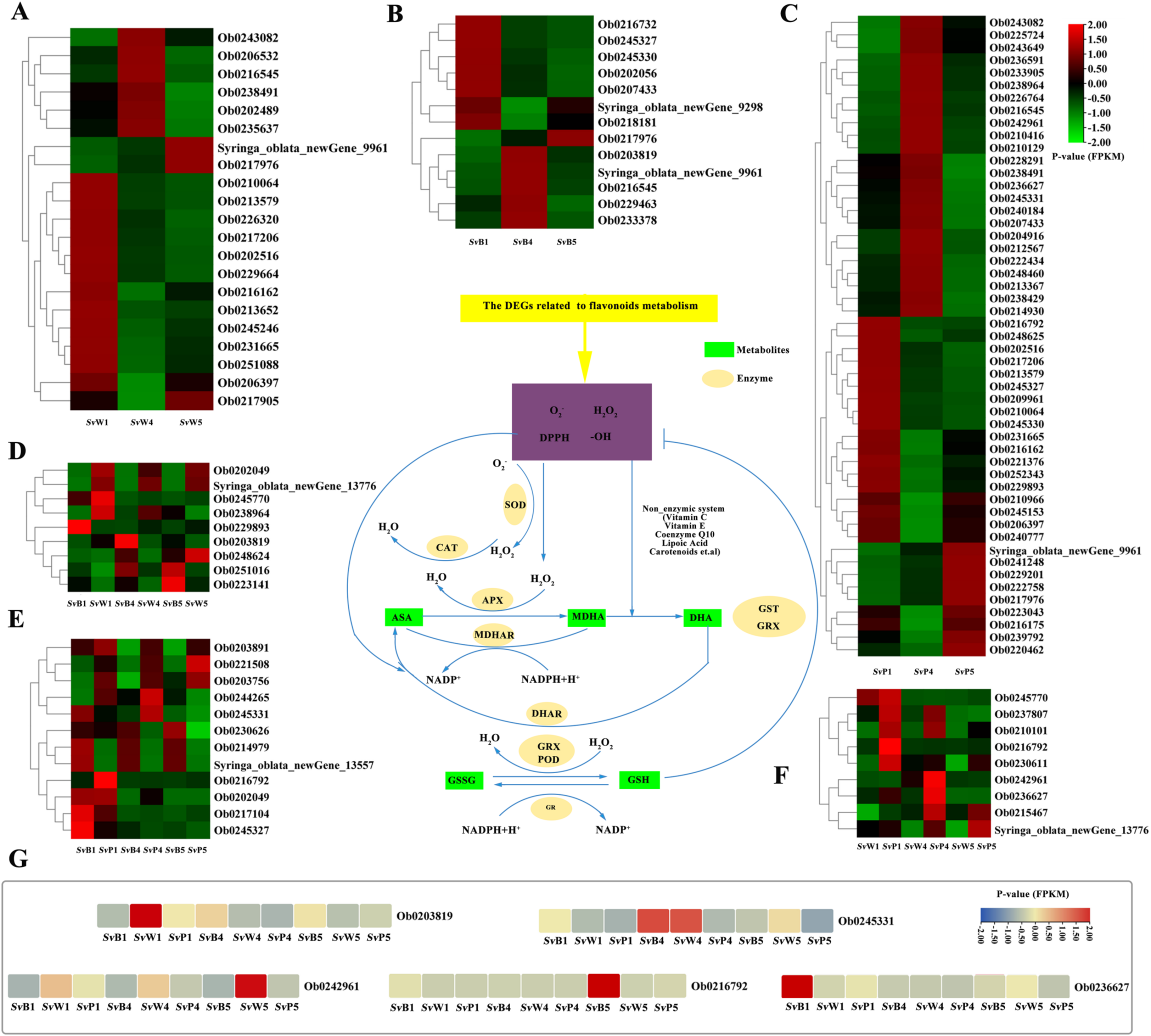
Analysis of DEGs related with antioxidant metabolism in three cultivars of *S. vulgaris.* **(A)** Heatmap of antioxidant-related DEGs present at developmental stages in *S. vulgaris* ‘Guifei III’ (W); **(B)** Heatmap of antioxidant-related DEGs present at all developmental stages in *S. vulgaris* ‘Wedgewood Blue’ **(B, C)** Heatmap of antioxidant-related DEGs present at developmental stages in *S. vulgaris* ‘Guifei IV’; **(D)** Heatmap of antioxidant-related DEGs between *S. vulgaris* ‘Wedgewood Blue’ and *S. vulgaris* ‘Guifei III’ present at developmental stages; **(E)** Heatmap of antioxidant-related DEGs between *S. vulgaris* ‘Wedgewood Blue’ and *S. vulgaris* ‘Guifei IV’ present at developmental stages; **(F)** DEGs between *S. vulgaris* ‘Guifei III’ and *S. vulgaris* ‘Guifei IV’ present at developmental stages; (**G**) Heatmap of antioxidant-related DEGs co-existing in genotypes and developmental stages of *S. vulgaris*.

We conducted a further analysis of the DEGs associated with antioxidant activity. In our gene-set-analysis groups, we identified 317 DEGs involved in the ascorbate-glutathione cycle and enzyme defense system (**Supplementary Table 10**; **Figure 5**). These genes encode key components of the enzymatic antioxidant defense system, including ascorbate peroxidase (*APX*), glutathione peroxidase (*GPX*), glutathione reductase (*GR)*, glutathione S-transferase (*GST*), dehydroascorbate reductase (*DHAR*), superoxide dismutase (*SOD*), peroxidase (*POD*), and catalase (*CAT*). We extracted the antioxidant-related DEGs in the B-d, W-d, and P-d gene-set-analysis groups, with 13, 22, and 22 DEGs, respectively (**Supplementary Table 10**; **Figures 5A-C**). These DEGs regulated the antioxidant system during the developmental stages of the three cultivars. The number of DEGs was significantly higher in the P-d group than in the B-d and W-d groups, indicating antioxidant system significantly changes at S*v*P cultivars’ development. The DEGs associated with antioxidant activity were observed, with 9, 12, and 9 DEGs identified in the BW-d, BP-d, and WP-d gene-set-analysis groups, respectively (**Supplementary Table 10**; **Figures 5D-F**). These DEGs collectively orchestrate genotype-specific modulation of the antioxidant defense system. Furthermore, the core set of 5 DEGs (*SvGST-1*, *SvGSTF11*, *SvPOD12*, *SvGR*, glutamate cysteine ligase gene (*SvGCL)*) was found to regulate both developmental and genotypic variations in antioxidant systems, highlighting their critical role in the antioxidant mechanism of *S. vulgaris* (**Supplementary Table 10**; **Figure 5G**).

### Integrative analysis in flavonoid-related metabolism

**Figure 6:**
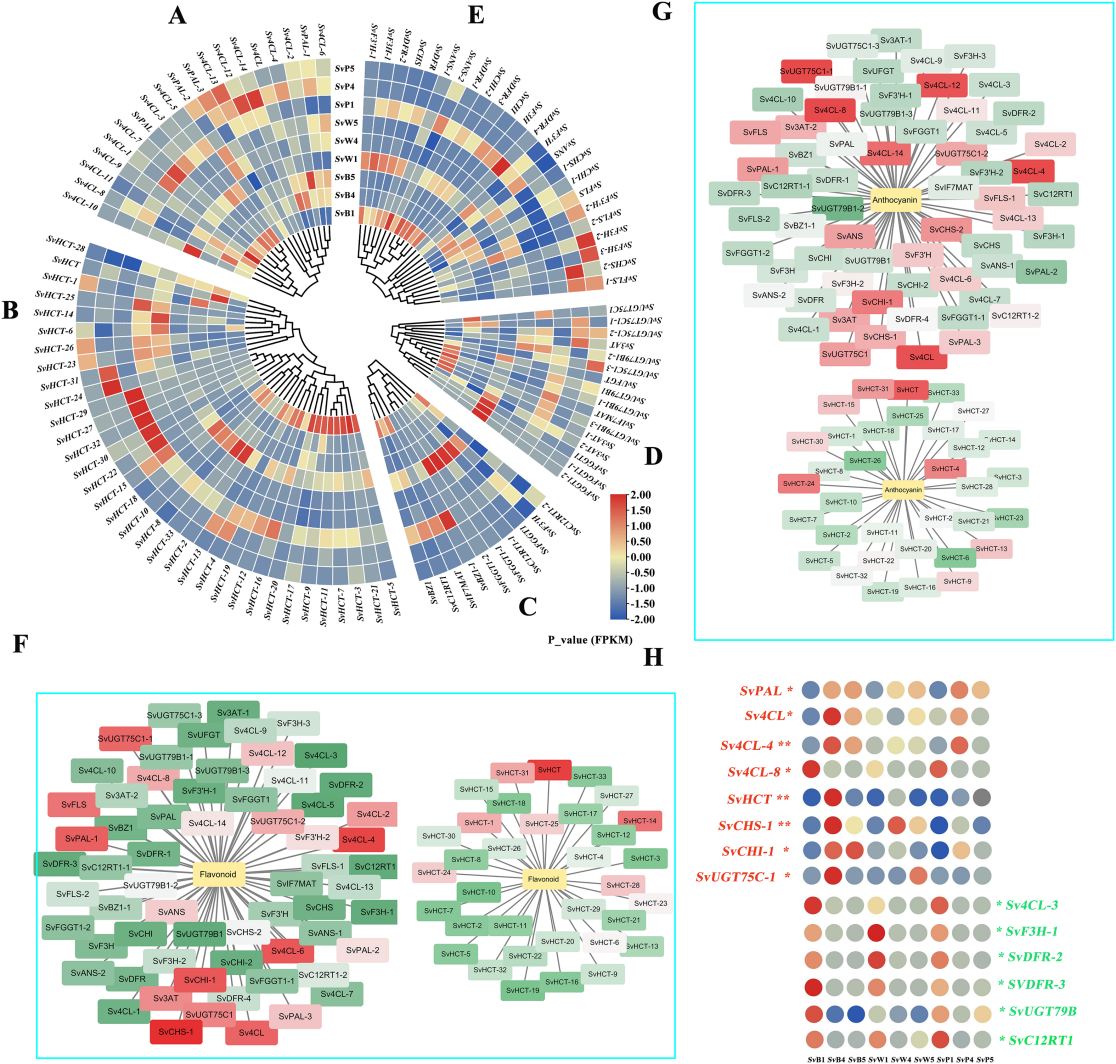
Integrated physiological and RNA-Seq analysis of flavonoid-related metabolism in three cultivars of *S. vulgaris.* **(A)** Heatmap of DEGs in phenylalanine metabolism; **(B)** Heatmap of DEGs in shikimate O-hydroxycinnamoyltransferase (HCT); **(C)** Heatmap of DEGs in flavone and flavonol biosynthesis; **(D)** Heatmap of DEGs in anthocyanin biosynthesis; **(E)** Heatmap of DEGs in flavonoids biosynthesis; **(F)** Correlation analysis of physiological and RNA-seq in flavonoid metabolism; **(G)** Correlation analysis of physiological and RNA-seq in anthocyanin metabolism; **(H)** Heatmap of DEGs that had higher correlation between physiological and RNA-seq analysis in flavonoid metabolism. The name of the red gene exhibits a significantly positive correlation. The name of the green gene exhibits a significantly negative correlation.

The structural DEGs that impact the metabolism of anthocyanins, flavones, and flavonols were identified from different gene-set-analysis groups (**Figures 6A-E**). Correlation analysis identified genes highly correlated with total flavonoid and anthocyanin content across three cultivars and stages (**Figures 6F, G**; **Supplementary Figure 3**; **Supplementary Table 11**). Among them, total flavonoid content was highly significant and positively correlated with the expression levels of *SvHCT*, SvCHS-1, and *Sv4CL-4* in the cultivars, while *SvPAL-1*, *Sv4CL-6*, and chalcone isomerase (*SvCHI-1*) also exhibited significant positive correlations with it. There were significant and negative correlations with total flavonoid content in the expression of *Sv4CL-3*, *Sv4CL-5*, *SvHCT-10*, *SvHCT-22*, *SvF3H-1*, *SvDFR-2*, *SvDFR-3*, flavanone 7-O-glucoside 2’’-O-beta-L-rhamnosyltransferase (*SvC12RT1*), and *SvUGT79B1*, *Sv3AT-1* in the cultivars. Our findings collectively demonstrate that these DEGs were pivotal regulators of flavonoid biosynthesis and accumulation in *S. vulgaris*, with *SvCHS-1* and *Sv4CL-4* emerging as particularly critical contributors to this metabolic process. Moreover, significant and positive correlations with total anthocyanin content were found in the *Sv4CL*, *Sv4CL-4*, *SvHCT*, *Sv4CL-8*, *Sv4CL-12*, and *SvUGT75C1-1*. They positively regulated the synthesis of anthocyanins in the three cultivars of *S. vulgaris*. Thus, the *Sv4CL-4*, *SvHCT*, *SvCHS-1*, *SvCHI-1*, *SvUGT75C1-1*, and *SvC12RT1* were core regulatory genes that directly governed flavonoid and anthocyanin content in the *S. vulgaris* analyze (**Figure 6H**; **Supplementary Figure 3**; **Supplementary Table 11**).

**Figure 7:**
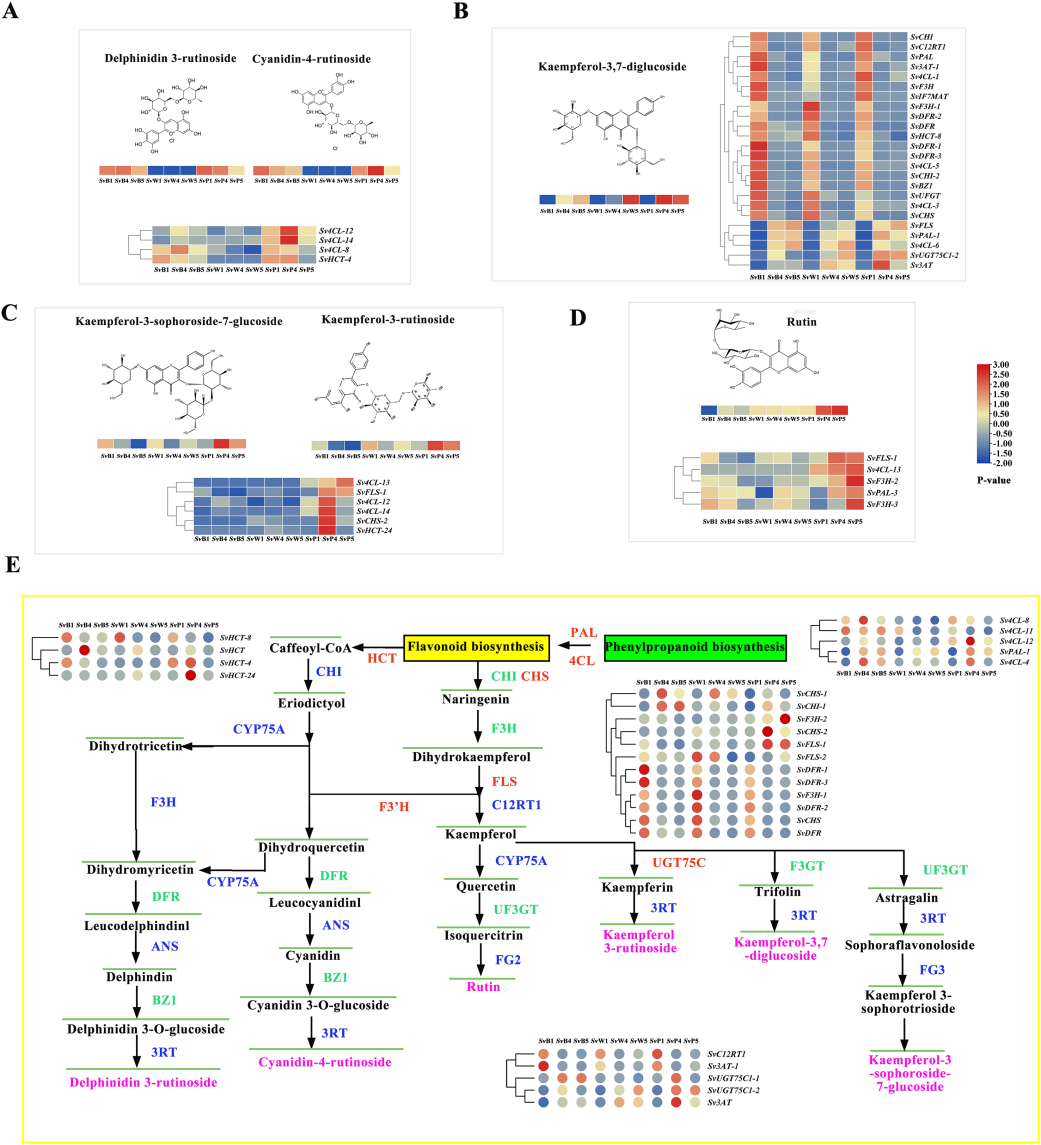
Integrated metabolomic and RNA-sequencing analysis of flavonoid-related metabolism in three cultivars of *S. vulgaris.* **(A)** Correlation analysis of DEGs related to flavonoid metabolism and Delphinidin-3-rutinoside and Cyanidin-4-rutinoside; **(B)** Correlation analysis of DEGs related to flavonoid metabolism and Kaempferol-3,7-diglucoside; **(C)** Correlation analysis of DEGs related to flavonoid metabolism and Kaempferol-3-rutinoside and Kaempferol-3-sophoroside-7-glucoside; **(D)** Correlation analysis of DEGs related to flavonoid metabolism and Rutin. **(E)** Integrated metabolomic and RNA-seq analysis in flavonoid-related metabolism of *S vulgaris*. The name of the red gene exhibits a significantly positive correlation. The name of the green gene exhibits a significantly negative correlation.

A correlation analysis was conducted between DEGs and metabolites of anthocyanins and flavonoids. The expression levels of *Sv4CL-8* and *Sv4CL-12* exhibited extremely significant and positive correlations with delphinidin-3-rutinoside and cyanidin-4-rutinoside, respectively (**Supplementary Figure 3**; **Supplementary Table 11**). The expression level of *SvHCT-4* exhibited extremely significant positive correlations with both delphinidin-3-rutinoside and cyanidin-4-rutinoside content (**Figure 7A**; **Supplementary Figure 3**; **Supplementary Table 11**), suggesting its potential role as a positive regulator in the biosynthesis of these anthocyanins and a key contributor to corolla pigmentation in *S. vulgaris*. In contrast, kaempferol-3,7-diglucoside accumulation was strongly positively correlated with the expression of *SvPAL-1*, *SvFLS*, and *SvUGT75C1-2*, while showing highly significant negative correlations with *SvHCT-8*, *SvCHS*, *SvCHI*, and *SvDFR* (**Figure 7B**; **Supplementary Figure 3**; **Supplementary Table 11**). Notably, a total of 15 structural genes and eight *HCT* family members were negatively associated with kaempferol-3,7-diglucoside levels. Furthermore, *SvCHS-2* and *SvFLS-1* expression was positively correlated with the accumulation of kaempferol-3-rutinoside and kaempferol-3-sophoroside-7-glucoside, respectively, highlighting their specialized roles in flavonol glycoside diversification (**Figure 7C**; **Supplementary Figure 3**; **Supplementary Table 11**). Meanwhile, the expression levels of *SvHCT-24* and *Sv4CL-14* showed highly significant and positive correlations with kaempferol-3-sophoroside-7-glucoside, while exhibited significant and positive correlations with kaempferol-3-rutinoside. The rutin maintained extremely significant and positive correlations with *Sv4CL-13*, S*vF3H-2*, and *SvFLS-1*, and also showed significant and positive correlations with the *SvPAL-3* and *SvF3H-3* (**Figure 7D**; **Supplementary Figure 3**; **Supplementary Table 11**). These findings indicated that specific DEGs might be implicated in the metabolic processes of four flavonoid derivatives.

Excavation and analysis identified a core set of genes essential for flavonoid synthesis in three cultivars, including *Sv4CL-4*, *SvHCT*, *SvHCT-4*, *SvCHS-1*, *SvCHI-1*, *SvUGT75C1-1*, and *SvC12RT1.* Among these, *SvHCT-4* specifically targets delphinidin-3-rutinoside and cyanidin-4-rutinoside, which were the primary pigments driving color variation in *S. vulgaris*. Subsequent metabolite correlation analysis revealed that *SvHCT*, *SvCHS-2*, and *SvFLS-1*, and others, directly regulate the biosynthesis of six key flavonoid compounds (**Figure 7E**).

### Integrative analysis in antioxidant-related pathways

**Figure 8:**
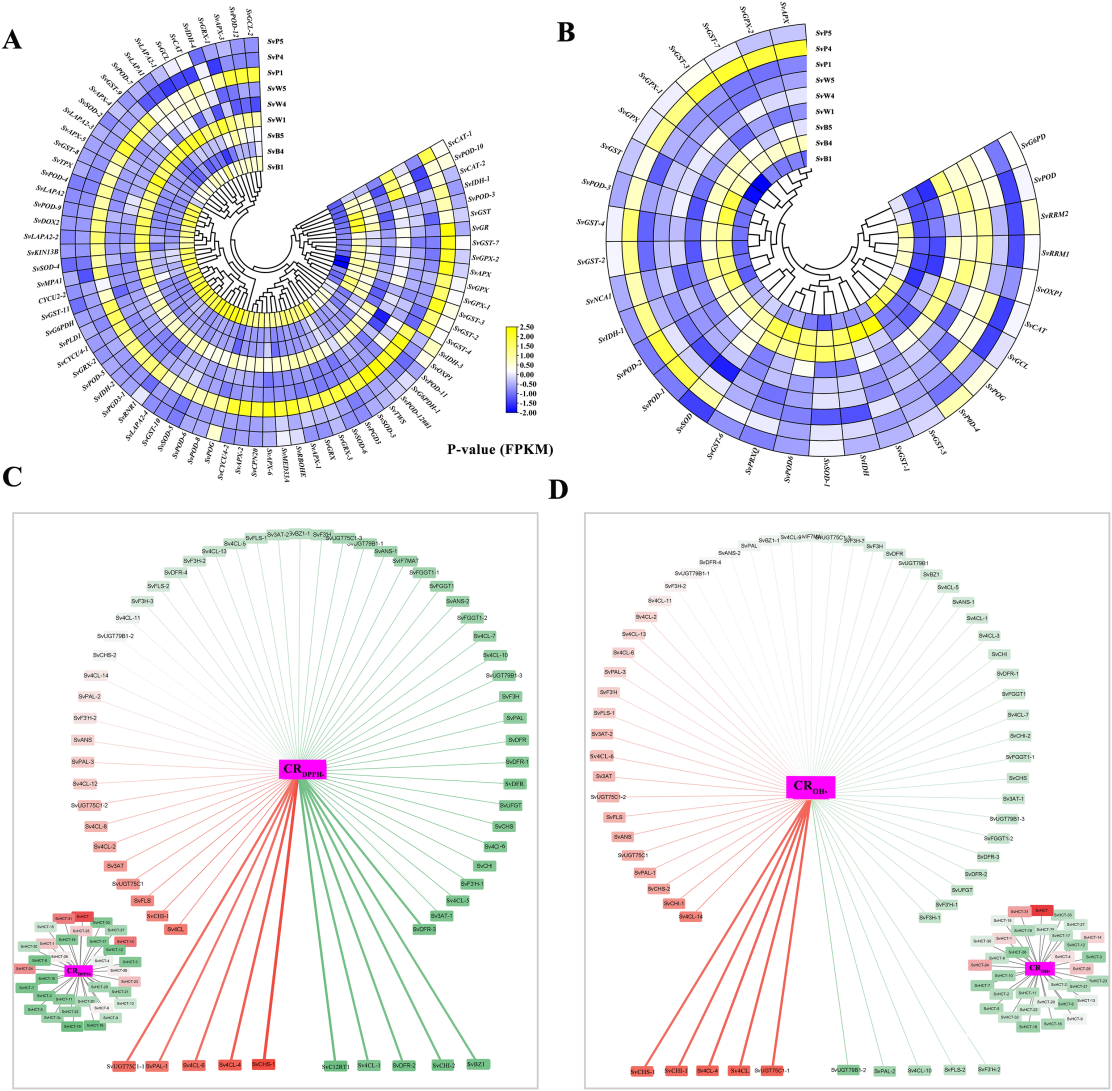
Multi-omics association analysis of antioxidant-related pathways in three cultivars of *S. vulgaris.* **(A)** Heatmap of antioxidant genes that have significant correlation with DPPH· scavenging rates in the three cultivars of *S. vulgaris*; **(B)** Heatmap of antioxidant genes that have significant correlation with OH·l scavenging rates in the three cultivars of *S. vulgaris*; **(C)** Correlation analysis between DEGs involved in flavonoid-related metabolism and DPPH· scavenging rates of *S. vulgaris*; **(D)** Correlation analysis of DEGs involved in flavonoid-related metabolism and OH· scavenging rates of *S. vulgaris.* CR_DPPH·_ and CR_OH·_ represent the clearance rates of 2,2-Diphenyl-1-picrylhydrazyl radical and hydroxyl radical.

**Figure 9:**
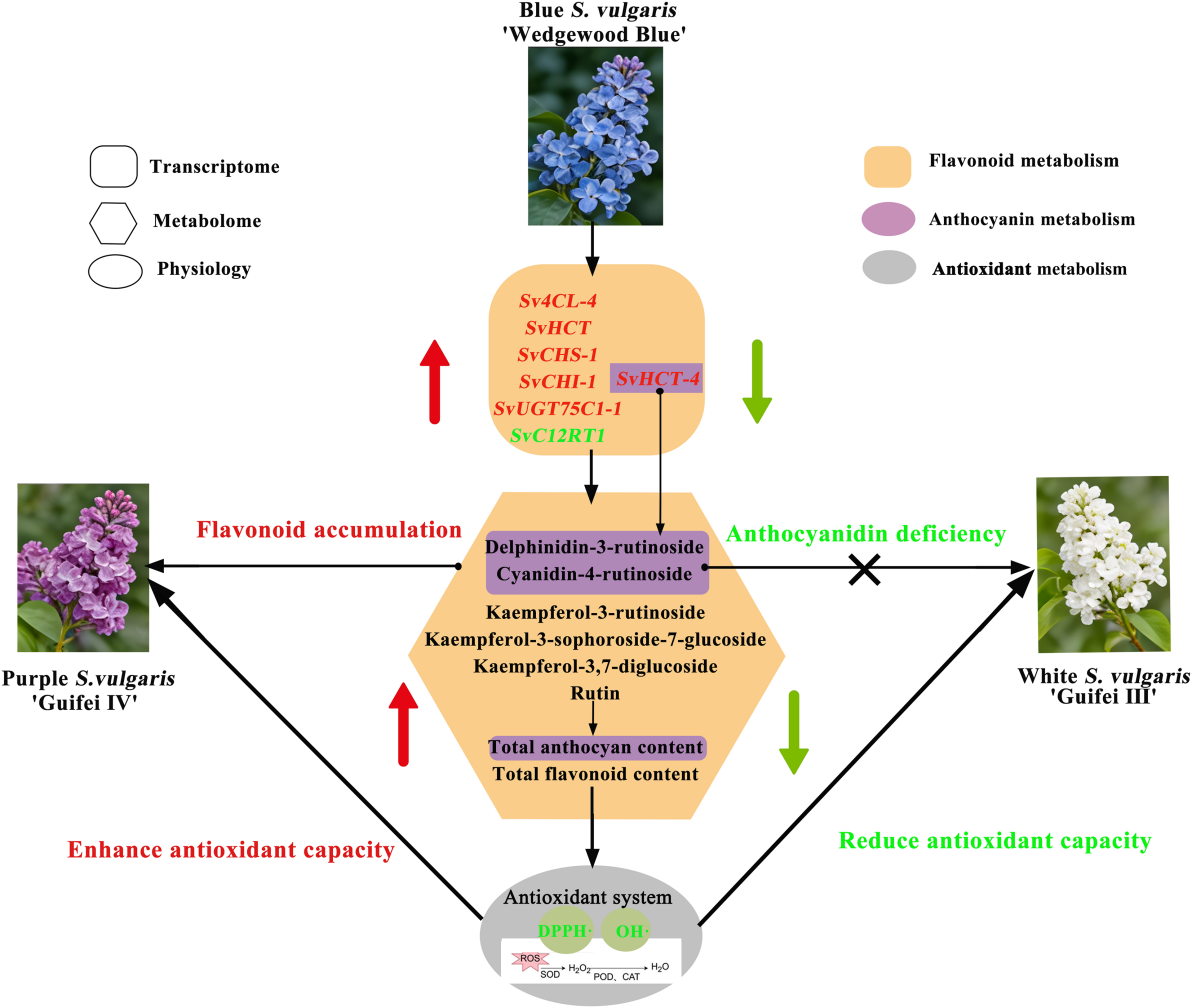
Molecular mechanism of flower color and antioxidant capacity in three cultivars of *S. vulgaris* until the full-bloom stages. The name of the red gene exhibits a significantly positive correlation. The name of the green gene exhibits a significantly negative correlation.

A strong association was observed between flavonoid metabolism and the antioxidant capacity in *S. vulgaris*. Comprehensive correlation analysis among the DEGs that were involved in flavonoids synthesis and scavenging rates of DPPH· and OH· in *S. vulgaris*. Notably, *Sv4CL-4*, *SvHCT*, *SvCHS-1*, and *SvC12RT1* exhibited extremely significant and positive correlations with the scavenging rate of DPPH· (**Supplementary Table 11**; **Figure 8C**). However, there were significant and negative correlations with scavenging rate of DPPH· in the expression levels of DEGs, including the *SvHCT-7*, *SvHCT-8*, *SvHCT-10*, *SvHCT-12*, *SvHCT-16*, and *SvHCT-19* (**Figure 8C**; **Supplementary Figure 3**; **Supplementary Table 11**). These genes may negatively regulate the DPPH· and OH· scavenging activities in the three cultivars of *S. vulgaris.* Furthermore, the expression levels of *Sv4CL-1*, *Sv4CL-4*, *SvHCT*, *SvCHI-1*, and *SvUGT75C1–1* also demonstrated extremely significant and positive correlations with the scavenging rate of OH· in the three cultivars. These outcomes further underscore the significant impact of these genes on the DPPH· and OH· scavenging activities within *S. vulgaris*. The expression levels of *Sv4CL-4*, *SvHCT*, *SvCHS-1*, *SvCHI-1*, and *SvUGT75C1–1* may positively regulate the flavonoid and anthocyanin synthesis to increase the DPPH· and OH· scavenging activities in the three cultivars (**Figure 9**).

Additionally, the correlation analysis between the scavenging rates of DPPH· and OH·, and DEGs in antioxidant-related pathways, and 74 and 31 DEGs that demonstrated significant correlations in the three cultivars of *S. vulgaris*, respectively (**Figures 8A, B**; **Supplementary Figure 3**; **Supplementary Table 11**). Our correlation analysis identified 18 and 14 DEGs with extremely significant correlations to DPPH· and OH· scavenging rates, respectively. Among these, isocitrate dehydrogenase genes (*SvIDH-4* and *SvIDH-1*) expression levels showed the strongest positive correlations with DPPH· and OH· scavenging rates, respectively. Conversely, *SvGCL* and *SvCAT* transcript levels exhibited the most significant negative correlations with these antioxidant metrics. We also observed highly significant positive correlations between *SvPOD-3*, *SvGST*, *SvGST-2*, *SvGPX-2* expression and both DPPH· and OH· scavenging rates. Conversely, there were highly significant and positive correlations between the specific activity of *SvCAT* in the three cultivars and the scavenging capacities of DPPH· and OH·. The findings indicated that the molecular antioxidant mechanisms in the three cultivars of *S. vulgaris* were substantially modified by these DEGs. These DEGs likely constitute pivotal regulatory nodes within the flavonoid-mediated antioxidant pathways, and need deep research.

## Discussion

### Two anthocyanin metabolites were key factors influencing floral pigmentation in cultivars of *S. vulgaris*

Flower color variation is a common phenomenon in nature, such as the changes from red, purple, or pink to yellow or white. Flower color transition from blue/purple to white is observed in *Ipomoea purpurea*, *Petunia hybrida*, *Parrya nudicauli*s, and *Torenia fournieri* (Dick et al., 2011; Morita et al., 2005; Nishijima et al., 2013; Zhang and Lu, 2016). The flower color of ornamental plants is determined by flavonoids and anthocyanins (Shen et al., 2024). In orchids, flavonoid content and specific pigment profiles that govern the transition from red to purple floral pigmentation (Li et al., 2020; Wang et al., 2011). The anthocyanins of different types and concentrations cause various flower colors (red, pink, and purple) in *Rosa*, *Pleione limprichtii*, and *Tulipa fosteriana* (Wan et al., 2019; Zhang et al., 2019; Yeon and Kim, 2020; Yuan et al., 2014). Flavonols and flavanols frequently serve as co-pigments of anthocyanins, and on certain occasions, they may impart a white hue to the blueberry (Iwashina, 2015; Sun et al., 2022). The delphinidin-3-O-rutinoside and cyanidin-3-O-rutinoside are key anthocyanins in the color metabolism of *S. oblata*, while cyanidin-3-O-glucoside and peonidin-3,5-di-O-glucoside regulate spotting in *Paeonia suffruticosa* (Gu et al., 2019; Ma et al., 2022). In the present study, a marked divergence in floral pigmentation was observed among the three cultivars of *S. vulgaris*, which directly correlated with dynamic fluctuations in total flavonoid and anthocyanin accumulation (**Figure 1**). This was consistent with blue petal coloration depending on anthocyanin synthesis and accumulation (Yoshida et al., 2009). The metabolomic profiling identified delphinidin-3-O-rutinoside and cyanidin-4-O-rutinoside as the dominant anthocyanins in the blue and purple flowered cultivars (*Sv*B and *Sv*P), with content strongly correlated to total anthocyanin levels (**Figure 9**). The absence of specific anthocyanin metabolites in white *S. vulgaris* cultivar caused the loss of intense purple-blue pigmentation, whereas their accumulation in colored cultivars directly determines the petal color phenotype. Cyanidin-derived anthocyanins, the driver of purple pigmentation in our study, exhibit the same patterns observed in *Jasminum officinale* and *Paeonia suffruticosa*, demonstrating this pathway is evolutionarily conserved across phylogenetically distant genera (Yang et al., 2020; Zhou et al., 2022).

Moreover, the accumulation and depletion of anthocyanins may be associated with gene expression in anthocyanin biosynthesis. The 4CL exhibits substrate specificity and its activity positively correlated to total anthocyanin and flavonol content in plant (Liu et al., 2021). Silencing of *CHS* in *Dahlia pinnata* and *Petunia hybrida* show white areas in petals (Morita et al., 2012; Ohno et al., 2011). As color of petals change from red to white, anthocyanin content and UFGT expression significantly decrease in *Nelumbo nucifera*, suggesting UFGT protein may be key to inlaid petal formation (Deng et al., 2023; Liu et al., 2022). Previous studies identified and functionally analyzed many genes linked to floral pigmentation in *S. oblata*, such as *CHS*, *C4H*, and *F3H* (Wang et al., 2016, 2018, 2017; Zheng et al., 2015). The HCT gene of *Physcomitrella patens* acts as a critical regulatory node that orchestrates the metabolic flux of the phenylpropanoid pathway throughout its reproductive developmental process, laying a foundational metabolic basis for the adaptation of early land plants to terrestrial environments (Kriegshauser et al., 2021). In three cultivars of *S. vulgaris*, flavonoid and anthocyanin content were regulated by a core set of 7 DEGs (*Sv4CL-4*, *SvHCT*, *SvHCT-4*, *SvCHS-1*, *SvCHI-1*, *SvUGT75C1-1*, and *SvC12RT1*) (**Figure 9**; **Supplementary Table 11**). This indicated that these genes may affect the floral pigmentation of *S. vulgaris* by regulating flavonoid and anthocyanin metabolites. Notably, *SvHCT-4* exhibited an extremely significant positive correlation with the contents of cyanidin-4-rutinoside and delphinidin-3-rutinoside (**Figure 8**). These results suggest that *SvHCT-4* likely contributes to fine-tuning the coloration of *S. vulgaris*. Previous studies on *HCT* genes mainly focused on lignin biosynthesis and stress response, with little attention to floral pigmentation. This study ‌hypothesized *SvHCT-4* as a positive regulator to key anthocyanins, filling a knowledge gap and offering new insights into petal color mechanisms in *S. vulgaris*. Further validation through over-expression or knockout could clarify its role and enable targeted pigment manipulation for horticulture.

Flavonoid biosynthesis is tightly governed by a network of transcription factors and environmental cues, with pigment transport and subcellular deposition further shaping the final petal color phenotype. WRKY and ERF transcription factors play distinct stage-specific roles in mediating flower color transitions of *S. oblata* (e.g., purplish-red to blue-purple, purple to light purple), while the MBW complex functions as a core regulator throughout the entire process of floral pigmentation (Ma et al., 2022). In lilac flower development, the lower expression of *SobHLH130* reduces *SoMYB44* transcripts and depresses transcriptional regulation of *SoUFGT1*, thus diminishing anthocyanin biosynthesis, leading to the fading of petal color (Wang et al., 2025). Transcription factors (PpBBX16, LvBBX24, and LvbZIP44) synergistically activate anthocyanidin biosynthesis genes to mediate light-responsive anthocyanidin accumulation in pear and petals of lilies (Bai et al., 2017; Gao Z. et al., 2024). Salt stress exerted an influence on the MYB5-ANR signaling pathway, leading to altered anthocyanins profiles in roses (Wang et al., 2024). Anthocyanin content dynamics were tightly coupled with *SvHCT-4* expression across developmental stages in three *S. vulgaris* cultivars (**Figure 8**; **Supplementary Table 11**). However, the precise regulatory function of *SvHCT-4* in flower color transitions awaits further investigation in the *S. vulgaris*. Comparative gene expression analysis will identify transcription factors that are co-expressed with *SvHCT-4* or target structure genes of flavonoids, and then clarify their roles in anthocyanin metabolism and flower color. The investigation of interactions between *SvHCT-4* and other enzymes may elucidate the basis of differential anthocyanin accumulation. Environmental factors, like light and temperature, should be considered for their interaction with regulatory pathways. Addressing these aspects can enhance understanding of flower color mechanisms in *S. vulgaris*, which can serve as a core reference for targeted breeding strategies toward desirable floral traits.

### Flavonoids modulated the antioxidant activity in cultivars of *S. vulgaris*

Flower color polymorphism is influenced by various pigment content, which increases the ornamental value of the plants and may have other physiological and ecological effects (Paine et al., 2019; Vaidya et al., 2018). The varying colors of roses exhibit distinct pigment compositions, which lead to differing antioxidant activities (Alizadeh and Fattahi, 2021). Similarly, the antioxidant potency of violas (*Viola* × *wittrockiana)* is influenced by their flower color (Ikeura et al., 2023). Flavonoids contribute to antioxidant capacity by scavenging hydroxyl radicals, superoxide anions, and DPPH radicals, and increased flavonol accumulation, which can enhance both antioxidant defense and drought tolerance in *Arabidopsis* (Nakabayashi et al., 2014). The chalcone, flavanone, procyanidins, isoflavone, and flavanols exhibit significant antioxidant activity in plants (Barreca et al., 2017; Gourlay et al., 2020; Sahu et al., 2012; Tian et al., 2014; Wen et al., 2013). The anthocyanins also exhibit extensive biological activities, such as anti-inflammatory and anticancer in rats and plants (Fang et al., 2022; Manzoor et al., 2022). Kaempferol-3-O-glucoside, the major metabolite in the leaf extract of *Erica multiflora*, showed high antioxidant activity and reduced the levels of pro-inflammatory cytokines (Khlifi et al., 2020). In this study, the total flavonoid content of the three cultivars of *S. vulgaris* were positively correlated with DPPH· and OH· scavenging abilities, aligning with previous research findings (**Supplementary Table 1**). The flavonoid content exerted a significant impact on the antioxidant properties of the three cultivars of *S. vulgaris*, and these properties varied among different cultivars and developmental stages. There were 22 and 5 flavonoid-related genes exhibited extremely significant correlations with DPPH· and OH· scavenging rates in the cultivars of *S. vulgaris*, respectively (**Supplementary Table 11**). The differential expression of these genes underpinned the observed variation in antioxidant activity across the three *S. vulgaris* cultivars at different stages. The stage-specific genetic modulation represents a viable strategy for enhancing their antioxidant capacity.

Antioxidant enzymes serve as a secondary line of defense in the antioxidant mechanism of plants. Under ROS stress, the activity of antioxidant enzymes, including SOD, CAT, DHAR, and APX, demonstrated an elevation (Zhu et al., 2021b). Eighteen and fourteen DEGs related to antioxidant enzymes in *S. vulgaris* plants showed strong correlations with DPPH· and OH· scavenging activities, respectively (**Supplementary Table 11**). The *SvPOD-3*, *SvGST*, *SvGST-2*, and *SvGPX-2* exhibited strong positive correlations with both DPPH· and OH· scavenging rates. These results further demonstrated that there were significant differences in the antioxidant activity of the three cultivars. The phenylpropanoids and glucosinolates are the two classes of secondary metabolites that can help plants survive during stress conditions (Kim et al., 2020). The flavonoids, such as luteolin, apigenin, and citrinin, display remarkable antioxidant properties through the suppression of pertinent gene expression, the stimulation of metabolic pathways, and the modulation of enzyme activity (Chagas et al., 2022; Heim et al., 2002). The significant correlations observed between these DEGs and the scavenging rates of DPPH· and OH· underscore their integral role in fortifying the plant’s antioxidant defense machinery against oxidative stress. Moreover, the flavonoid and anthocyanin metabolites may act as upstream regulators, fine-tuning antioxidant capacity through the transcriptional modulation of key genes that encode antioxidant enzymes in *S. vulgaris.* These findings elucidated the molecular basis of oxidative stress resilience in *S. vulgaris* and provided a genetic and metabolic framework for breeding programs to enhance its antioxidant potential for nutritional and pharmaceutical uses.

Numerous regulators associated with flavonoids and antioxidant activity have been identified across various plants in previous studies (Shen et al., 2022). The six flavonoid metabolites were detected in three *S. vulgaris* cultivars but showed no significant correlation with DPPH· and OH· clearance rates. The result indicated no direct influence between the flavonoid metabolites and antioxidant activity. This discrepancy may stem from several factors. First, flavonoids mediate antioxidant effects in concert with co-occurring phytochemicals through cooperative redox interactions (Varga et al., 2019). Second, DPPH· and OH· *in vitro* assays may not fully reflect *in vivo* mechanisms, where flavonoids may up-regulate cellular defense systems rather than directly scavenge radicals (Heim et al., 2002). Additionally, variations in flavonoid glycosylation or methylation could reduce their solubility, limiting reactivity under assay conditions (Tian et al., 2024). In this study, we characterized flavonoid-related and antioxidant-related genes that demonstrated significant correlations with the scavenging of DPPH^-^ and OH^-^ radicals. Future studies will focus on these genes, and will conduct regulatory mechanism analysis to further validate the functional roles of these genes. Subsequent investigations on the synergistic relationships between these six anthocyanin and flavonoid metabolites and other plant endogenous substances will expose novel antioxidant mechanisms that remain invisible in single-metabolite detection workflows.

### Metabolic flux partitioning of the flavonoids coordinates floral pigmentation and antioxidant activity in cultivars of *S. vulgaris*

As major plant secondary metabolites, flavonoids including anthocyanins contribute both vibrant pigmentation and robust antioxidant activity. The anthocyanin content is generally closely related to the antioxidant capacity in black soybeans (Hashimoto et al., 2015). The different colors of potatoes exhibit varying antioxidant activities, and this activity significantly increases with the enhancement of anthocyanin content (Lachman et al., 2009; Xiao et al., 2016). Heterologous expression of snapdragon transcription factors Delila and Rosea1 in tomato fruit drove massive anthocyanin accumulation, conferring intense purple pigmentation and a threefold enhancement of antioxidant capacity (Butelli et al., 2008). The *GbMYBF2* expression is negatively correlated with flavonoid accumulation in *Ginkgo biloba leaves*, and inhibits gene expressions such as *F3H*, *CHS*, *FLS*, and *ANS*, significantly reducing quercetin, kaempferol, and anthocyanin content (Xu et al., 2014). However, when flavonoid metabolism is perturbed, anthocyanin biosynthesis and non-anthocyanin sometimes flavonoids display fundamentally distinct regulatory trajectories. For instance, ectopic expression of the *FLS* gene from *Camellia sinensis* promotes the accumulation of kaempferol and reduced the anthocyanin content of flowers in tobacco (Jiang et al., 2020). When ectopically expressed genes of *Meconopsis integrifolia* in tobacco, *MiDFR6* directed dihydromyricetin into the delphinidin branch of the anthocyanin pathway, while *MiFLS2* siphoned the shared dihydroflavonol substrate toward flavonol synthesis (Ou et al., 2026). In the study, all three cultivars exhibited synchronized peaks in flavonoid accumulation, anthocyanin biosynthesis, and antioxidant capacity at the half-open and full-flowering stages. The result revealed a conserved temporal coordination of secondary metabolite dynamics across genotypes (**Figure 1**). This temporal congruence supported a shared regulatory framework governing both floral pigmentation and antioxidant capacity. However, the white cultivar was devoid of detectable anthocyanins, which retained the antioxidant activity induced by non-anthocyanin flavonoids. This finding demonstrated that anthocyanin biosynthesis and antioxidant activity by non-anthocyanin flavonoids are governed by distinct regulatory trajectories. Flavonoids orchestrated both floral pigmentation and antioxidant capacity in *S. vulgaris*, yet their co-regulation was not mediated by a unified pathway. The metabolic flux partitioning mechanisms of flavonoids is required to elucidate the exact mechanisms and fully exploit their potential.

However, few studies systematically investigated the underlying metabolic flux partitioning mechanism responsible for the biosynthesis and regulation of flavonoids, leaving a significant gap in our understanding of this critical biochemical pathway. In the study, the flavonoid metabolism of *S. vulgaris* was regulated by a core set of the 7 DEGs including *SvHCT-4.* Meanwhile, the SvHCT-4 redirected metabolic flux from the general phenylpropanoid pathway to anthocyanin biosynthesis, thereby enhancing the accumulation of cyanidin-4-O-rutinoside and delphinidin-3-O-rutinoside in the blue and purple cultivars *S. vulgaris* (**Figures 8**, **9**). These findings revealed competitive yet coordinated mechanisms governing metabolic flux partitioning of flavonoids, which impact on floral pigmentation and antioxidant activity in *S. vulgaris*. HCT functions as a key regulatory node that that partitions p-coumaroyl-CoA flux between anthocyanin and lignin/flavonol biosynthesis by catalyzing its esterification with shikimate or quinate, thereby governing floral coloration and antioxidant capacity. Recent evidence shows HCT acts as a metabolic gatekeeper, with its expression and substrate affinity balancing pigments and flavonols to influence developmental signaling and environmental adaptation (Gao Y. et al., 2024). The HCT-encoding gene *SvHCT-4* regulated the homeostasis of flavonoid and anthocyanin biosynthesis of *S. vulgaris*, while influencing the antioxidant activity of flavonoid derivatives. These mechanisms were manifested not only in the interdependent constraints across distinct metabolic pathways but also in the precise modulation of metabolic flux through differential expression of genes encoding key enzymes. This multi-level regulatory network ultimately determines the specific combination of flower color phenotypes and antioxidant capacity across different cultivars.

Since other factors also affect floral pigmentation and antioxidant properties, a comprehensive and in-depth analysis of specific regulatory mechanisms is still needed. Further studies are warranted to explore the interactions between these identified genes and metabolites, as well as their roles in response to environmental factors, to fully elucidate the complex mechanisms underlying the floral pigmentation and antioxidant properties of *S. vulgaris* flowers. Future research should focus on elucidating environmental factors such as light, temperature, and soil conditions to explore their interactions with specific genes and enzymes that are involved in flavonoid biosynthesis in *S. vulgaris*. Additionally, further elucidation of the applications of these flavonoids across horticultural breeding, pharmaceutical development, and nutritional science may yield fundamental insights into their biological roles while revealing their commercial promise.

## Conclusion

In conclusion, this research had significantly advanced our understanding of the molecular and metabolic underpinnings of flower color and antioxidant activity in three different color cultivars of *Syringa vulgaris*. By employing a multi-omics approach, we had identified key metabolites and genes involved in flavonoid and anthocyanin biosynthesis, elucidating the genetic and biochemical pathways that contribute to the observed phenotypic variations. First, spatiotemporal dynamics of total flavonoid and anthocyanin accumulation in the *S. vulgaris* across genotypes and developmental stages were tightly coupled to floral pigmentation and radical scavenging capacities. Second, six flavonoid and anthocyanin metabolites were profiled in floral tissues, and cyanidin-4-rutinoside served as the primary determinant of inter-cultivar variation in total anthocyanin accumulation, directly shaping both petal chromaticity and radical scavenging efficacy. Third, RNA sequencing identified 95 and 317 key genes affecting flavonoid metabolism and antioxidant mechanisms, and 11 DEGs were crucial for flavonoids. Finally, multi-omics analysis showed that a core set of genes (*Sv4CL-4*, *SvHCT*, *SvHCT-4*, *SvCHS-1*, *SvCHI-1*, *SvUGT75C1-1*, and *SvC12RT1*) modulated flavonoid metabolism, impacting flower color and antioxidant mechanisms. The *SvHCT-4* orchestrated floral pigmentation in *S. vulgaris* by selectively enhancing the biosynthesis of cyanidin-4-rutinoside and delphinidin-3-rutinoside. These flavonoids and anthocyanidins activated the antioxidant metabolism, and led to the DPPH· and OH· scavenging capabilities varying significantly in the *S. vulgaris*. This study constituted a substantial advancement in our pursuit of comprehending and regulating the metabolic flux partitioning related to flower color and antioxidant activity in *S. vulgaris*. It provided a reliable framework for subsequent investigations focused on improving the ornamental and health-promoting characteristics of this well-favored plant species.

## Data Availability

The original contributions presented in the study are publicly available. This data can be found here: China National Center for Bioinformation (CNCB), CRA044441.

## Supplementary material

Supplementary Figure 1 KEGG enrichment of DEGs between developmental stages in three cultivars of *S. vulgaris.* **(A)** Venn diagram of differential genes (DEGs) in different groups **(B)** Dotplot of DEGs between developmental stages in *S. vulgaris* ‘Wedgewood Blue’; **(C)** Dotplot of DEGs between developmental stages in *S. vulgaris* ‘Guifei III’; **(D)** Dotplot of DEGs between developmental stages in *S. vulgaris* ‘Guifei IV’. *Sv*B: S. vulgaris ‘Wedgewood Blue’; *Sv*W: the white *S. vulgaris* ‘Guifei III’; *Sv*P: the purple *S. vulgaris* ‘Guifei IV’.

Supplementary Figure 2 KEGG enrichment of DEGs between three cultivars of *S. vulgaris.* **(A)** Venn diagram of differential genes (DEGs) in different groups; **(B)** Dotplot of DEGs between *S. vulgaris* ‘Wedgewood Blue’ and *S. vulgaris* ‘Guifei III’; **(C)** Dotplot of DEGs between *S. vulgaris* ‘Wedgewood Blue’ and *S. vulgaris* ‘Guifei IV’; **(D)** Dotplot of DEGs between *S. vulgaris* ‘Guifei III’ and *S. vulgaris* ‘Guifei IV’.

Supplementary Figure 3 Multi-omics association analysis in three cultivars of *S. vulgaris*.

Supplementary Table 1 Correlation Analysis between physiological indicators and metabolomic profiles in three cultivars of *S. vulgaris.*

Supplementary Table 2 Content and relative content of flavonoids and anthocyanins in the metabolomic analysis of three cultivars of *S. vulgaris.*

Supplementary Table 3 The quality analysis of all reads from all samples in RNA-seq analysis.

Supplementary Table 4 Mappedstat in RNA-seq analysis.

Supplementary Table 5 All_Database_annotation in RNA-seq analysis.

Supplementary Table 6 Information of Gene-Set-Analysis groups in RNA-seq analysis.

Supplementary Table 7 DEGs analysis.

Supplementary Table 8 KEGG pathway enrichment.

Supplementary Table 9 DEGs related to the flavonoid metabolic pathway in different comparison combinations.

Supplementary Table 10 Candidate DEGs analysis.

Supplementary Table 11 Candidate DEGs correlation analysis.

## References

[B1] Alizadeh Z. Fattahi M. (2021). Essential oil, total phenolic, flavonoids, anthocyanins, carotenoids and antioxidant activity of cultivated Damask Rose (Rosa damascena) from Iran: With chemotyping approach concerning morphology and composition. Sci. Hortic. 288, 110341. doi: 10.1016/j.scienta.2021.110341 38826717

[B2] Bai S. Sun Y. Qian M. Yang F. Ni J. Tao R. . (2017). Transcriptome analysis of bagging-treated red Chinese sand pear peels reveals light-responsive pathway functions in anthocyanin accumulation. Sci. Rep. 7, 63. doi: 10.1038/s41598-017-00069-z 28246400 PMC5428347

[B3] Barreca D. Gattuso G. Bellocco E. Calderaro A. Trombetta D. Smeriglio A. . (2017). Flavanones: Citrus phytochemical with health-promoting properties. Biofactors 43, 495–506. doi: 10.1002/biof.1363 28497905

[B4] Bueno J. M. Ramos-Escudero F. Sáez-Plaza P. Muñoz A. M. José Navas M. Asuero A. G. (2012). Analysis and antioxidant capacity of anthocyanin pigments. Part I: General considerations concerning polyphenols and flavonoids. Crit. Rev. Anal. Chem. 42, 102–125. doi: 10.1080/10408347.2011.632312 37339054

[B5] Butelli E. Licciardello C. Zhang Y. Liu J. Mackay S. Bailey P. . (2012). Retrotransposons control fruit-specific, cold-dependent accumulation of anthocyanins in blood oranges. Plant Cell 24, 1242–1255. doi: 10.1105/tpc.111.095232 22427337 PMC3336134

[B6] Butelli E. Titta L. Giorgio M. Mock H. P. Matros A. Peterek S . (2008). Enrichment of tomato fruit with health-promoting anthocyanins by expression of select transcription factors. Nat. Biotechnol. 26, 1301–1308. doi: 10.1038/nbt.1506 18953354

[B7] Chagas M. Behrens M. D. Moragas-Tellis C. J. Penedo G. X. M. Silva A. R. Gonçalves-de-Albuquerque C. F. (2022). Flavonols and flavones as potential anti-inflammatory, antioxidant, and antibacterial compounds. Oxid. Med. Cell. Longev. 2022, 9966750. doi: 10.1155/2022/9966750 36111166 PMC9470311

[B8] Chen L. Xia B. Li Z. Liu X. Bai Y. Yang Y. . (2022). Syringa oblata genome provides new insights into molecular mechanism of flower color differences among individuals and biosynthesis of its flower volatiles. Front. Plant Sci. 13, 1078677. doi: 10.3389/fpls.2022.1078677 36618636 PMC9811319

[B9] Dai S. Hong Y. (2016). Molecular breeding for flower colors modification on ornamental plants based on the mechanism of anthocyanins biosynthesis and coloration. Scientia Agricultura Sin. 49, 14. doi: 10.3864/j.issn.0578-1752.2016.03.011 41470978

[B10] Deeva A. M. Shabunya P. S. Fatykhova S. A. Zubarev A. V. Reshetnikov V. N. Spiridovich E. V. (2022). Anthocyanin content in the flowers of common lilac varieties (Syringa vulgaris L.). Russ. J. Plant Physiol. 69, 26. doi: 10.1134/S1021443722020042

[B11] Deng J. Su M. Zhang X. Liu X. Damaris R. N. Lv S. . (2023). Proteomic and metabolomic analyses showing the differentially accumulation of NnUFGT2 is involved in the petal red-white bicolor pigmentation in lotus (Nelumbo nucifera). Plant Physiol. Biochem. 198, 107675. doi: 10.1016/j.plaphy.2023.107675 37043997

[B12] Dick C. A. Buenrostro J. Butle T. Carlson M. L. Kliebenstein D. J. Whittall J. B. (2011). Arctic mustard flower color polymorphism controlled by petal-specific downregulation at the threshold of the anthocyanin biosynthetic pathway. PloS One 6, e18230. doi: 10.1371/journal.pone.0018230 21490971 PMC3072389

[B13] El-Desouky S. K. Gamal-Eldeen A. M. (2009). Cytotoxic and anti-inflammatory activities of some constituents from the flower buds of Syringa patula. Pharm. Biol. 47, 872–877. doi: 10.1080/13880200902950785 37339054

[B14] Fang Z. Luo Y. Ma C. Dong L. Chen F. (2022). Blueberry anthocyanins extract attenuates acrylamide-induced oxidative stress and neuroinflammation in rats. Oxid. Med. Cell. Longev. 2022, 7340881. doi: 10.1155/2022/7340881 35651724 PMC9151000

[B15] Forkmann G. (1991). Flavonoids as flower pigments the formation of the natural spectrum and its extension by genetic engineering. Plant Breed. 106, 1–26. doi: 10.1111/j.1439-0523.1991.tb00474.x 40046247

[B16] Gao Y. W. Huan Z. G. Zhang Z. Y. He L. Huang Z. J. Chen J. W. . (2024). UDP-glucosyltransferase 71C4 controls the flux of phenylpropanoid metabolism to shape cotton seed development. Plant Commun. 5, 725. doi: 10.1016/j.xplc.2024.100938 38689494 PMC11369780

[B17] Gao Z. Sun Y. Zhu Z. Ni N. Sun S. Nie M. . (2024). Transcription factors LvBBX24 and LvbZIP44 coordinated anthocyanin accumulation in response to light in lily petals. Hortic. Res. 11, uhae211. doi: 10.1093/hr/uhae211 39372289 PMC11450212

[B18] Gąsecka M. Krzymińska-Bródka A. Magdziak Z. Czuchaj P. Bykowska J. (2023). Phenolic compounds and organic acid composition of Syringa vulgaris L. flowers and infusions. Molecules 28, 5159. doi: 10.3390/molecules28135159 37446821 PMC10343234

[B19] Gourlay G. Ma D. Schmidt A. Constabel C. P. (2020). MYB134-RNAi poplar plants show reduced tannin synthesis in leaves but not roots, and increased susceptibility to oxidative stress. J. Exp. Bot. 71, 6601–6611. doi: 10.1093/jxb/eraa371 32777037

[B20] Grotewold E. (2006). The genetics and biochemistry of flower pigments. Annu. Rev. Plant Biol. 57, 761–780. doi: 10.1146/annurev.arplant.57.032905.105248 16669781

[B21] Gu Z. Zhu J. Hao Q. Yuan Y. W. Duan Y. W. Men S. . (2019). A novel R2R3-MYB transcription factor contributes to petal blotch formation by regulating organ-specific expression of PsCHS in tree peony (Paeonia suffruticosa). Plant Cell Physiol. 60, 599–611. doi: 10.1093/pcp/pcy232 30496505

[B22] Han M. Zhao Y. Meng J. Yin J. Li H. (2023). Analysis of physicochemical and antioxidant properties of Malus spp. petals reveals factors involved in flower color change and market value. Sci. Hortic. 310, 111688. doi: 10.1016/j.scienta.2022.111688 38826717

[B23] Hanganu D. Niculae M. Ielciu I. Olah N. K. Munteanu M. Burtescu R. . (2021). Chemical profile, cytotoxic activity and oxidative stress reduction of different Syringa vulgaris L. extracts. Molecules 26, 3104. doi: 10.3390/molecules26113104 34067400 PMC8197011

[B24] Hashimoto N. Oki T. Sasaki K. Suda I. Okuno S. (2015). Black soybean seed coat extract prevents hydrogen peroxide-mediated cell death via extracellular signal-related kinase signalling in HepG2 cells. J. Nutr. Sci. Vitaminol (Tokyo) 61, 275–279. doi: 10.3177/jnsv.61.275 26226966

[B25] Heim K. E. Tagliaferro A. R. Bobilya D. J. (2002). Flavonoid antioxidants: chemistry, metabolism and structure-activity relationships. J. Nutr. Biochem. 13, 572–584. doi: 10.1016/s0955-2863(02)00208-5 12550068

[B26] Hoffmann L. Besseau S. Geoffroy P. Ritzenthaler C. Meyer D. Lapierre C. . (2004). Silencing of hydroxycinnamoyl-coenzyme A shikimate/quinate hydroxycinnamoyltransferase affects phenylpropanoid biosynthesis. Plant Cell 16, 1446–1465. doi: 10.1105/tpc.020297 15161961 PMC490038

[B27] Houghton A. Appelhagen I. Martin C. (2021). Natural blues: Structure meets function in anthocyanins. Plants (Basel) 10, 726. doi: 10.3390/plants10040726 33917946 PMC8068391

[B28] Ikeura H. Kobayashi F. Kai T. Tsuchiya Y. Tamaki M. (2023). Flower colour and antioxidant activity of violas (Viola × wittrockiana) as edible flowers. J. Hortic. Sci. Biotechnol. 98, 678–684. doi: 10.1080/14620316.2023.2170833 37339054

[B29] Iwashina T. (2015). Contribution to flower colors of flavonoids including anthocyanins: a review. Nat. Prod. Commun. 10, 529–544. doi: 10.1177/1934578x1501000335 25924543

[B30] Jędrzejuk A. Rabiza-Świder J. Skutnik E. Serek M. (2016). Flowering conditions affect flower longevity in Syringa vulgaris and cause changes in protein content, protease activity and expression of a KDEL-CysEP gene. Acta Physiol. Plant 38, 45. doi: 10.1007/s11738-015-2044-z 30311153

[B31] Jakubczyk K. Koprowska K. Gottschling A. Janda-Milczarek K. (2022). Edible flowers as a source of dietary fibre (total, insoluble and soluble) as a potential athlete's dietary supplement. Nutrients 14, 2407. doi: 10.3390/nu14122470 35745200 PMC9231144

[B32] Ji P. Xue B. Guo J. Li X. (2012). Physiological changes of Syringa L. during the flowering and senescence. J. Inner Mongolia Forestry Sci. Technol. 38, 27–29. doi: 10.3969/j.issn.1007-4066.2012.01.007

[B33] Jiang X. Shi Y. Fu Z. Li W. W. Lai S. Wu Y. . (2020). Functional characterization of three flavonol synthase genes from Camellia sinensis: Roles in flavonol accumulation. Plant Sci. 300, 110632. doi: 10.1016/j.plantsci.2020.110632 33180711

[B34] Khlifi R. Dhaouefi Z. Toumia I. B. Lahmar A. Sioud F. Bouhajeb R. . (2020). Erica multiflora extract rich in quercetin-3-O-glucoside and kaempferol-3-O-glucoside alleviates high fat and fructose diet-induced fatty liver disease by modulating metabolic and inflammatory pathways in Wistar rats. J. Nutr. Biochem. 86, 108490. doi: 10.1016/j.jnutbio.2020.108490 32920086

[B35] Kim M. J. Shin H. S. (2012). Antioxidative effect of lotus seed and seedpod extracts. Food Sci. Biotechnol. 21, 1761–1766. doi: 10.1007/s10068-012-0234-7 30311153

[B36] Kim J. I. Zhang X. Pascuzzi P. E. Liu C. J. Chapple C. (2020). Glucosinolate and phenylpropanoid biosynthesis are linked by proteasome-dependent degradation of PAL. New Phytol. 225, 154–168. doi: 10.1111/nph.16108 31408530

[B37] Knoch E. Sugawara S. Mori T. Nakabayashi R. Saito K. Yonekura-Sakakibara K. (2018). UGT79B31 is responsible for the final modification step of pollen-specific flavonoid biosynthesis in Petunia hybrida. Planta-Berlin. 247 (4), 779–790. doi: 10.1007/S00425-017-2822-5 29214446 PMC5856881

[B38] Kriegshauser L. Knosp S. Grienenberger E. Tatsumi K. Gütle D. D. Sørensen I. . (2021). Function of the HYDROXYCINNAMOYL-CoA:SHIKIMATE HYDROXYCINNAMOYL TRANSFERASE is evolutionarily conserved in embryophytes. Plant Cell 33, 1472–1491. doi: 10.1093/plcell/koab044 33638637 PMC8254490

[B39] Kurkin V. A. Zapesochnaya G. G. Grinenko N. A. Zolotarev B. M. (1989). Phenolic compounds of the bark of *Syringa vulgaris*. Chem. of Nat. Compounds 25, 499–500. doi: 10.1007/bf00597667

[B40] Lachman J. Hamouz K. Šulc M. Orsák M. Pivec V. Hejtmánková A. . (2009). Cultivar differences of total anthocyanins and anthocyanidins in red and purple-fleshed potatoes and their relation to antioxidant activity. Food Chem. 114, 836–843. doi: 10.1016/j.foodchem.2008.10.029 38826717

[B41] Lepiniec L. Debeaujon I. Routaboul J.-M. Baudry A. Pourcel L. Nesi N. . (2006). Genetics and biochemistry of seed flavonoids. Annu. Rev. Plant Biol. 57, 405–430. doi: 10.1146/annurev.arplant.57.032905.105252 16669768

[B42] Li B. J. Zheng B. Q. Wang J. Y. Tsai W. C. Lu H. C. Zou L. H. . (2020). New insight into the molecular mechanism of colour differentiation among flower segments in orchids. Commun. Biol. 3, 89. doi: 10.1038/s42003-020-0821-8 32111943 PMC7048853

[B43] Liu W. Feng Y. Yu S. Fan Z. Li X. Li J. . (2021). The flavonoid biosynthesis network in plants. Int. J. Mol. Sci. 22, 12824. doi: 10.3390/ijms222312824 34884627 PMC8657439

[B44] Liu H. Su B. Zhang H. Gong J. Zhang B. Liu Y. . (2019). Identification and functional analysis of a flavonol synthase gene from grape hyacinth. Molecules 24, 1579. doi: 10.3390/molecules24081579 31013599 PMC6514955

[B45] Liu J. Wang Y. Zhang M. Wang Y. Deng X. Sun H. . (2022). Color fading in lotus (Nelumbo nucifera) petals is manipulated both by anthocyanin biosynthesis reduction and active degradation. Plant Physiol. Biochem. 179, 100–107. doi: 10.1016/j.plaphy.2022.03.021 35325657

[B46] Ma S. Wang M. Li P. Guo L. Xiong L. Tian Y. . (2024). Transcriptome and metabolome analysis reveal the lip color variation in Cymbidium floribundum. Ornamental Plant Res. 4, e019. doi: 10.48130/opr-0024-0017

[B47] Ma B. Wu J. Shi T. L. Yang Y. Y. Wang W. B. Zheng Y. . (2022). Lilac (Syringa oblata) genome provides insights into its evolution and molecular mechanism of petal color change. Commun. Biol. 5, 686. doi: 10.1038/s42003-022-03646-9 35810211 PMC9271065

[B48] Manolescu B. N. Oprea E. Mititelu M. Ruta L. L. Farcasanu I. C. (2019). Dietary anthocyanins and stroke: a review of pharmacokinetic and pharmacodynamic studies. Nutrients 11, 1479. doi: 10.3390/nu11071479 31261786 PMC6682894

[B49] Manzoor M. F. Hussain A. Naumovski N. Ranjha M. Ahmad N. Karrar E. . (2022). A narrative review of recent advances in rapid assessment of anthocyanins in agricultural and food products. Front. Nutr. 9, 901342. doi: 10.3389/fnut.2022.901342 35928834 PMC9343702

[B50] Morita Y. Saito R. Ban Y. Tanikawa N. Kuchitsu K. Ando T. . (2012). Tandemly arranged chalcone synthase A genes contribute to the spatially regulated expression of siRNA and the natural bicolor flower phenotype in Petunia hybrida. Plant J. 70, 739–749. doi: 10.1111/j.1365-313X.2012.04908.x 22288551

[B51] Morita Y. Tanaka Y. Kikuchi Y. Nitasaka E. Hoshino A. Saito N. . (2005). Japanese morning glory dusky mutants displaying reddish-brown or purplish-gray flowers are deficient in a novel glycosylation enzyme for anthocyanin biosynthesis, UDP-glucose: anthocyanidin 3-O-glucoside-2''-O-glucosyltransferase, due to 4-bp insertions in the gene. Plant J. 42, 353–363. doi: 10.1111/j.1365-313X.2005.02383.x 15842621

[B52] Nakabayashi R. Yonekura-Sakakibara K. Urano K. Suzuki M. Yamada Y. Nishizawa T. . (2014). Enhancement of oxidative and drought tolerance in Arabidopsis by overaccumulation of antioxidant flavonoids. Plant J. 77, 367–379. doi: 10.1111/tpj.12388 24274116 PMC4282528

[B53] Nishijima T. Morita Y. Sasaki K. Nakayama M. Yamaguchi H. Ohtsubo N. . (2013). A Torenia (Torenia fournieri Lind. ex Fourn.) novel mutant ‘Flecked’ produces variegated flowers by insertion of a DNA transposon into an R2R3-MYB gene. J. Japanese Soc. For. Hortic. Sci. 82, 39–50. doi: 10.2503/jjshs1.82.39

[B54] Ohno S. Hosokawa M. Kojima M. Kitamura Y. Hoshino A. Tatsuzawa F. . (2011). Simultaneous post-transcriptional gene silencing of two different chalcone synthase genes resulting in pure white flowers in the octoploid dahlia. Planta 234, 945–958. doi: 10.1007/s00425-011-1456-2 21688014

[B55] Okitsu N. Matsui K. Horikawa M. Sugahara K. Tanaka Y. (2018). Identification and characterization of novel Nemophila menziesii flavone glucosyltransferases that catalyze biosynthesis of flavone 7,4 '-O-diglucoside, a key component of blue metalloanthocyanins. Plant Cell Physiol. 59 (10), 2075–2085. doi: 10.1093/pcp/pcy129 29986079

[B56] Ou Z. Tan C. Zhou L. Wang H. Liang S. Liu Z. . (2026). Multilayer Regulation of Yellow Flower Pigmentation in Meconopsis integrifolia by Competing Enzymes MiFLS2 and MiDFR6. Plant, Cell Environ. 49, 2362–2374. doi: 10.1111/pce.70384 41531315

[B57] Paine K. C. White T. E. Whitney K. D. (2019). Intraspecific flower color variation as perceived by pollinators and non-pollinators: evidence for pollinator-imposed constraints? Evol. Ecol. 33, 461–479. doi: 10.1007/s10682-019-09991-2 30311153

[B58] Peng J. Dong X. Xue C. Liu Z. Cao F. (2021). Exploring the molecular mechanism of blue flower color formation in Hydrangea macrophylla cv. "Forever Summer. Front. Plant Sci. 12, 585665. doi: 10.3389/fpls.2021.585665 33679822 PMC7925886

[B59] Petit D. P. Teppa E. Harduin-Lepers A. (2020). A phylogenetic view and functional annotation of the animal β1,3-glycosyltransferases of the GT31 CAZy family. Glycobiology. 31 (3), 243–259. doi: 10.1093/glycob/cwaa086 32886776 PMC8022947

[B60] Punyasiri P. A. N. Abeysinghe I. S. B. Kumar V. Treutter D. Duy D. Gosch C. . (2004). Flavonoid biosynthesis in the tea plant Camellia sinensis: properties of enzymes of the prominent epicatechin and catechin pathways. Arch. Biochem. Biophys. 431, 22–30. doi: 10.1016/j.abb.2004.08.003 15464723

[B61] Qi Q. Chu M. Yu X. Xie Y. Li Y. Du Y. . (2023). Anthocyanins and proanthocyanidins chemical structures food sources bioactivities and product development. Food Rev. Int. 39, 4581–4609. doi: 10.1080/87559129.2022.2029479 37339054

[B62] Qian R. Ye Y. Ma X. Gao H. Hu Q. Zheng J. (2025). Targeted metabolome and transcriptome analysis reveals the key metabolites and genes influencing blue–purple colour development in Clematis lanuginosa flowers. Ornamental Plant Res. 5, e001. doi: 10.48130/opr-0024-0031

[B63] Ramsay N. A. Glover B. J. (2005). MYB-bHLH-WD40 protein complex and the evolution of cellular diversity. Trends Plant Sci. 10, 63–70. doi: 10.1016/j.tplants.2004.12.011 15708343

[B64] Sahu N. K. Balbhadra S. S. Choudhary J. Kohli D. V. (2012). Exploring pharmacological significance of chalcone scaffold: a review. Curr. Med. Chem. 19, 209–225. doi: 10.2174/092986712803414132 22320299

[B65] Shen Y. Rao Y. Ma M. Li Y. He Y. Wang Z. . (2024). Coordination among flower pigments, scents and pollinators in ornamental plants. Horticulture Adv. 2, 6. doi: 10.1007/s44281-024-00029-4 30311153

[B66] Shen N. Wang T. Gan Q. Liu S. Wang L. Jin B. (2022). Plant flavonoids: Classification, distribution, biosynthesis, and antioxidant activity. Food Chem. 383, 132531. doi: 10.1016/j.foodchem.2022.132531 35413752

[B67] Sun X. Shokri S. Gao B. Xu Z. Li B. zhu T. . (2022). Improving effects of three selected co-pigments on fermentation, color stability, and anthocyanins content of blueberry wine. Food Science and Technology. 156, 113070. doi: 10.1016/j.lwt.2022.113070 38826717

[B68] Tian F. Jia T. Yu B. (2014). Physiological regulation of seed soaking with soybean isoflavones on drought tolerance of Glycine max and Glycine soja. Plant Growth Regul. 74, 229–237. doi: 10.1007/s10725-014-9914-z 30311153

[B69] Tian Q. Y. Xu W. Guang C. E. Zhang W. L. Mu B. W. (2024). Glycosylation of flavonoids by sucrose- and starch-utilizing glycoside hydrolases: A practical approach to enhance glycodiversification. Crit. Rev. Food Sci. Nutr. 64, 7408–7425. doi: 10.1080/10408398.2023.2185201 36876518

[B70] Tóth G. Barabás C. Tóth A. Kéry Á. Béni S. Boldizsár I. . (2016). Characterization of antioxidant phenolics in Syringa vulgaris L. flowers and fruits by HPLC-DAD-ESI-MS. Biomed. Chromatogr. 30, 923–932. doi: 10.1002/bmc.3630 26433204

[B71] Trapnell C. Williams B. A. Pertea G. Mortazavi A. Kwan G. van Baren M. J. . (2010). Transcript assembly and quantification by RNA-Seq reveals unannotated transcripts and isoform switching during cell differentiation. Nat. Biotechnol. 28, 511–515. doi: 10.1038/nbt.1621 20436464 PMC3146043

[B72] Vaidya P. McDurmon A. Mattoon E. Keefe M. Carley L. Lee C. R. . (2018). Ecological causes and consequences of flower color polymorphism in a self-pollinating plant (Boechera stricta). New Phytol. 218, 380–392. doi: 10.1111/nph.14998 29369384

[B73] Varga E. Barabás C. Tóth A. Boldizsár I. Noszál B. Tóth G. (2019). Phenolic composition, antioxidant and antinociceptive activities of Syringa vulgaris L. bark and leaf extracts. Nat. Prod. Res. 33, 1664–1669. doi: 10.1080/14786419.2018.1425855 29336171

[B74] Wan H. Yu C. Han Y. Guo X. Luo L. Pan H. . (2019). Determination of flavonoids and carotenoids and their contributions to various colors of rose cultivars (Rosa spp.). Front. Plant Sci. 10, 123. doi: 10.3389/fpls.2019.00123 30809238 PMC6379320

[B75] Wang Y. Dou Y. Wang R. Guan X. Hu Z. Zheng J. (2017). Molecular characterization and functional analysis of chalcone synthase from Syringa oblata Lindl. in the flavonoid biosynthetic pathway. Gene 635, 16–23. doi: 10.1016/j.gene.2017.09.002 28890377

[B76] Wang Q. Du B. Bai Y. Chen Y. Li F. Du J. . (2024). Saline-alkali stress affects the accumulation of proanthocyanidins and sesquiterpenoids via the MYB5-ANR/TPS31 cascades in the rose petals. Hortic. Res. 11, uhae243. doi: 10.1093/hr/uhae243 39534410 PMC11554761

[B77] Wang Y. Li Z. Sun H. Gouo H. Zhang G. (2011). Molecular mechanism of flower color formation and variation in orchids. Mol. Plant Breed. (Online) 9, 1584–1590. doi: 10.5376/mpb.cn.2011.09.0080

[B78] Wang Y. Lu L. Li J. Li H. Yi C. Y. Zang S. (2022). A chromosome-level genome of syringa oblata provides new insights into chromosome formation in oleaceae and evolutionary history of lilacs. Plant Journal: Cell. Mol. Biol. 111 (3), 836–848. doi: 10.1111/tpj.15858 35673966

[B79] Wang R. Ren C. Dong S. Chen C. Xian B. Wu Q. . (2021). Integrated metabolomics and transcriptome analysis of flavonoid biosynthesis in safflower (Carthamus tinctorius L.) with different colors. Front. Plant Sci. 12, 712038. doi: 10.3389/fpls.2021.712038 34381487 PMC8351732

[B80] Wang J. W. W. X. Ma B. Leng P. S. Wu B. J. Hu Z. H. (2025). SoNAC72-SoMYB44/SobHLH130 module contributes to flower color fading via regulating anthocyanin biosynthesis by directly binding to the SoUFGT1 promoter in lilac (Syringa oblata). Hortic. Res. 12, uhae326. doi: 10.1093/hr/uhae326 40046329 PMC11879506

[B81] Wang R. Wang Y. Li Y. Zheng J. (2016). Cloning and expression analysis of cinnamic acid 4-hydroxylase gene from Syringa oblata Lindl. Mol. Plant Breed. 14 (8), 2025–2030. doi: 10.13271/j.mpb.014.002025 42185099

[B82] Wang R. Zheng J. Li Y. Xie Y. (2018). Cloning and expression analysis of flavanone 3-hydroxylase gene from Syringa oblata Lindl. Mol. Plant Breed. 16 (6), 3863–3869. doi: 10.13271/j.mpb.016.003863

[B83] Wen P. Niu X. Xing Y. Niu T. Gao M. Ji Z. . (2013). The effect of UV-C irradiation on spatial and temporal accumulation of flavanols and the activity, tissue localization of LAR in grape berry. Acta Hortic. Sin. 40, 1251–1261. doi: 10.3864/j.issn.0578-1752.2012.21.011 42185099

[B84] Xia Z. Fan W. Liu D. Chen Y. Lv J. Xu M. . (2024). Haplotype-resolved chromosomal-level genome assembly reveals regulatory variations in mulberry fruit anthocyanin content. Hortic. Res. 11. doi: 10.1093/hr/uhae120 38919559 PMC11197311

[B85] Xia X. Gong R. Zhang C. (2022). Integrative analysis of transcriptome and metabolome reveals flavonoid biosynthesis regulation in rhododendron pulchrum petals. BMC Plant Biol. 22, 1–20. doi: 10.1186/s12870-022-03762-y 35974307 PMC9380304

[B86] Xiao J. Yang X. Guo H. (2016). Antioxidant activities of anthocyanins from 'Jianchuanhong' and 'Zhuanxinwu' pigmented potatoes. Food. Sci. 37 (13), 13–18. doi: 10.7506/spkx1002-6630-201613003

[B87] Xu F. Ning Y. Zhang W. Liao Y. Li L. Cheng H. . (2014). An R2R3-MYB transcription factor as a negative regulator of the flavonoid biosynthesis pathway in Ginkgo biloba. Funct. Integr. Genomics 14, 177–189. doi: 10.1007/s10142-013-0352-1 24306138

[B88] Yamasaki H. Uefuji H. Sakihama Y. (1996). Bleaching of the red anthocyanin induced by superoxide radical. Arch. Biochem. Biophys. 332, 183–186. doi: 10.1006/abbi.1996.0331 8806724

[B89] Yang C. Li H. Zhu J. Han M. Shen T. Meng J. (2020). Analysis of petal pigment and related gene expression during flower color change of Paeonia ostii ‘feng dan'. J. Northeast. Forestry Univ. 48, 62–66. doi: 10.3969/j.issn.1000-5382.2020.05.012

[B90] Yeon J. Y. Kim W. S. (2020). Flower pigment-scent associations in eight cut rose cultivars with various petal colors. Hortic. Environ. Biotechnol. 61, 633–641. doi: 10.1007/s13580-020-00249-3 30311153

[B91] Yiğit R. Çoklar H. Akbulut M. (2022). Some physicochemical and phytochemical properties of Syringa vulgaris L. flower tea: influence of flower drying technique, brewing method and brewing time. J. Food Meas. Charact. 16, 4185–4197. doi: 10.1007/s11694-022-01511-1 30311153

[B92] Yoshida K. Mori M. Kondo T. (2009). Blue flower color development by anthocyanins: from chemical structure to cell physiology. Nat. Prod. Rep. 26, 884–915. doi: 10.1039/b800165k 19554240

[B93] Yuan Y. Qiang S. Ma X. Tang D. Gu J. Shi Y. (2014). Anthocyanin compositions and changes in Tulipa fosteriana 'Shangnong Zaoxia'. J. Shanghai Jiaotong Univ. (Agricultural Science). 32 (3), 8. doi: 10.3969/J.ISSN.1671-9964.2014.03.013

[B94] Yuan Y. W. Rebocho A. B. Sagawa J. M. Stanley L. E. Bradshaw H. D. (2016). Competition between anthocyanin and flavonol biosynthesis produces spatial pattern variation of flower pigments between Mimulus species. Proc. Natl. Acad. Sci. U.S.A. 113, 2448–2453. doi: 10.1073/pnas.1515294113 26884205 PMC4780602

[B95] Zhang H. Gong J. Chen K. Yao W. Du L. (2020). A novel r3 myb transcriptional repressor, mamybx, finely regulates anthocyanin biosynthesis in grape hyacinth. Plant Sci. 298, 110588. doi: 10.1016/j.plantsci.2020.110588 32771147

[B96] Zhang R. Lu Y. (2016). Molecular mechanisms and natural selection of flower color variation. Botanical Res. 5, 186–209. doi: 10.12677/BR.2016.56024

[B97] Zhang L. Shan Y. Tang K. Putheti R. (2009). Ultrasound-assisted extraction flavonoids from Lotus (Nelumbo nuficera Gaertn) leaf and evaluation of its anti-fatigue activity. Int. J. Phys. Sci. 4, 418–422. doi: 10.1142/S0218127409024505 31116912

[B98] Zhang Y. Zhou T. Dai Z. Dai X. Li W. Cao M. . (2019). Comparative transcriptomics provides insight into floral flower color polymorphism in a Pleione limprichtii orchid population. Int. J. Mol. Sci. 21, 247. doi: 10.3390/ijms21010247 31905846 PMC6982098

[B99] Zhao M. Wang S. Chen L. Zhang J. Yao Y. Tian J. (2024). Transcriptomic analyses reveal the effects of grafting on anthocyanin biosynthesis in crabapple. Ornamental Plant Res. 4, e021. doi: 10.48130/opr-0024-0018

[B100] Zheng J. Hu Z. Guan X. Dou D. Bai G. Wang Y. . (2015). Transcriptome analysis of Syringa oblata Lindl. inflorescence identifies genes associated with pigment biosynthesis and scent metabolism. PloS One 10, e0142542. doi: 10.1371/journal.pone.0142542 26587670 PMC4654506

[B101] Zhou L. G. Feng X. S. Huang K. Y. He L. Deng X. M. Wang D. C. (2008). Studies on chemical constituents of Syringa veutina. Zhong Yao Cai 31, 679–681. doi: 10.3321/j.issn:1001-4454.2008.05.019 18826143

[B102] Zhou M. Jiang M. Ying X. Cui Q. Han Y. Hou Y. . (2013). Identification and comparison of anti-inflammatory ingredients from different organs of Lotus Nelumbo by UPLC/Q-TOF and PCA coupled with a NF-κB reporter gene assay. PloS One 8. doi: 10.1371/journal.pone.0081971 24312388 PMC3843740

[B103] Zhou B. Yan X. Lei Y. Peng C. Chen L. (2022). Variation of flower color and pigment contents of Mirabilis jalapa during flowering. Bull. Botanical Res. 42, 475–482. doi: 10.7525/j.issn.1673-5102.2022.03.017

[B104] Zhu W. B. Wang Z. B. Sun Y. P. Yang B. Y. Wang Q. H. Kuang H. X. (2021a). Traditional uses, phytochemistry and pharmacology of genus Syringa: a comprehensive review. J. Ethnopharmacol. 266, 113465. doi: 10.1016/j.jep.2020.112988 33049343

[B105] Zhu K. J. Zheng X. J. Ye J. L. Huang Y. Chen H. Y. Mei X. H. . (2021b). Regulation of carotenoid and chlorophyll pools in hesperidia, anatomically unique fruits found only in Citrus. Plant Physiol. 187, 829–845. doi: 10.1093/plphys/kiab291 34608960 PMC8491056

