# Supplementary figures and images for: Integrative analysis of the metabolome and transcriptome reveals the mechanism of flower color and antioxidant capacity in three Syringa cultivars
Source: Front Plant Sci. 2026 Jul 3;17:1840931. doi: 10.3389/fpls.2026.1840931 (PMC13375870; doi:10.3389/fpls.2026.1840931)

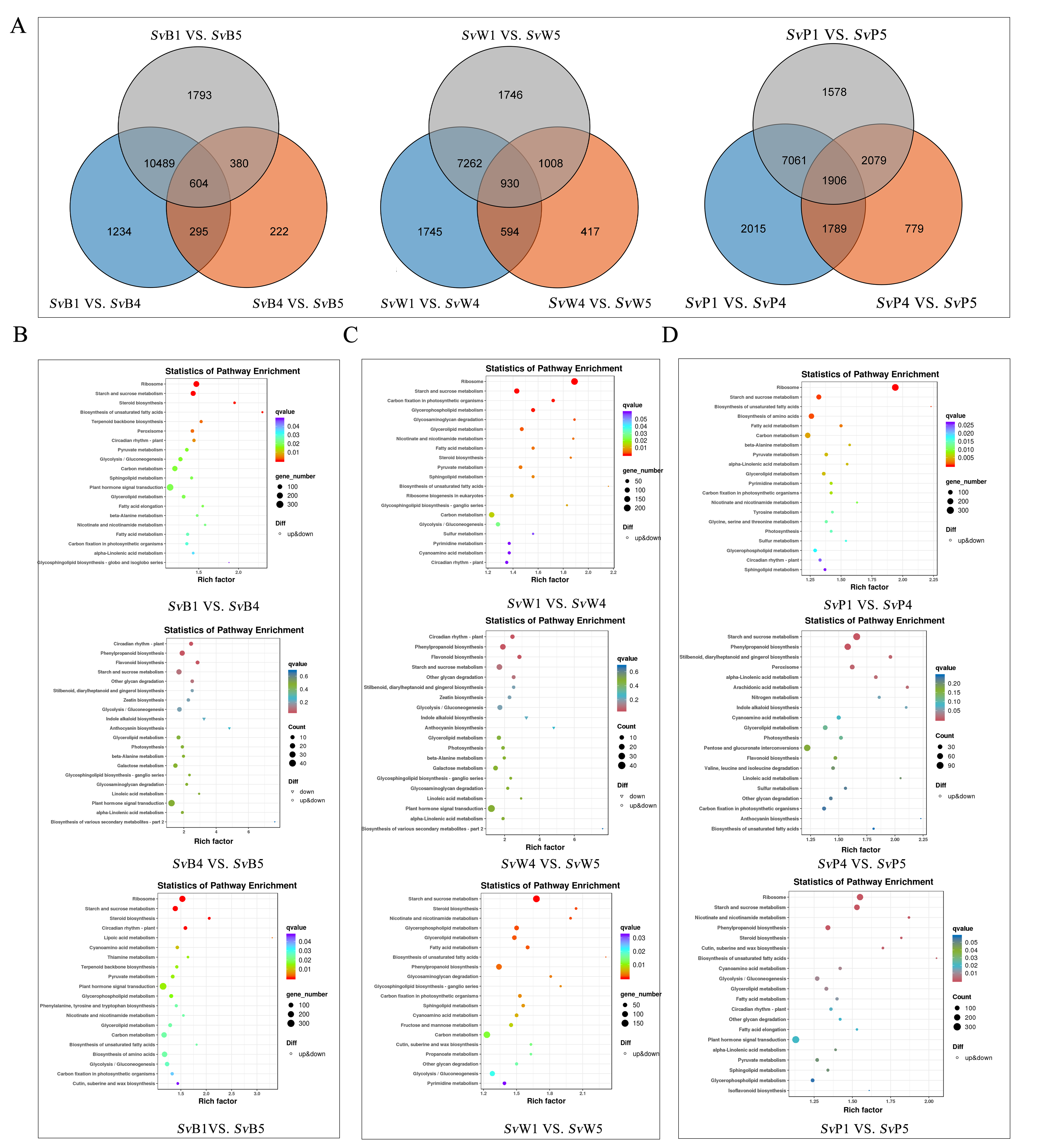

Supplement: Supplementary Figure 1 — KEGG enrichment of DEGs between developmental stages in three cultivars of S. vulgaris. (A) Venn diagram of differential genes (DEGs) in different groups (B) Dotplot of DEGs between developmental stages in S. vulgaris ‘Wedgewood Blue’; (C) Dotplot of DEGs between developmental stages in S. vulgaris ‘Guifei III’; (D) Dotplot of DEGs between developmental stages in S. vulgaris ‘Guifei IV’. SvB: S. vulgaris ‘Wedgewood Blue’; SvW: the white S. vulgaris ‘Guifei III’; SvP: the purple S. vulgaris ‘Guifei IV’. [file Image1.tif]

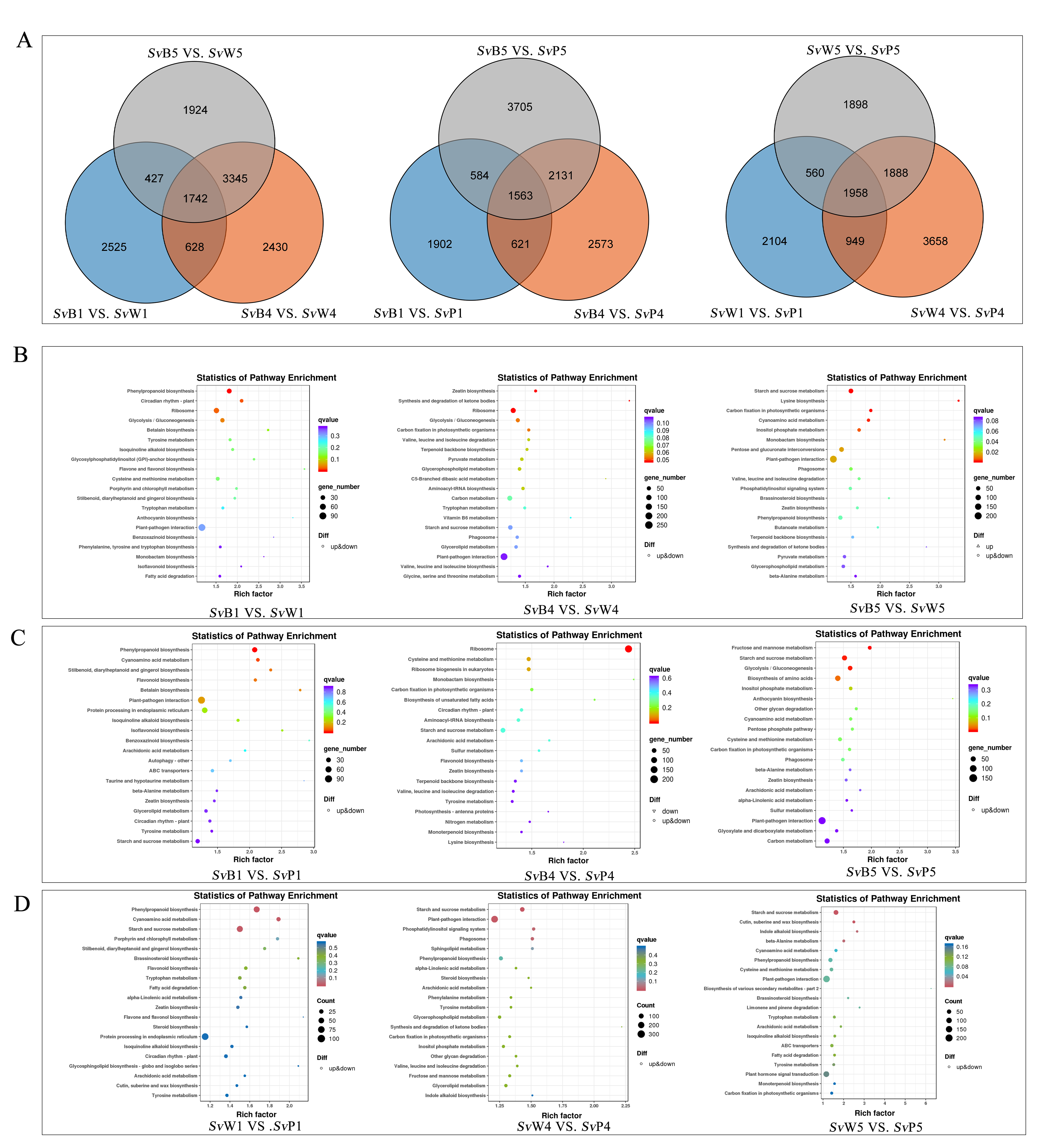

Supplement: Supplementary Figure 2 — KEGG enrichment of DEGs between three cultivars of S. vulgaris. (A) Venn diagram of differential genes (DEGs) in different groups; (B) Dotplot of DEGs between S. vulgaris ‘Wedgewood Blue’ and S. vulgaris ‘Guifei III’; (C) Dotplot of DEGs between S. vulgaris ‘Wedgewood Blue’ and S. vulgaris ‘Guifei IV’; (D) Dotplot of DEGs between S. vulgaris ‘Guifei III’ and S. vulgaris ‘Guifei IV’. [file Image2.tif]

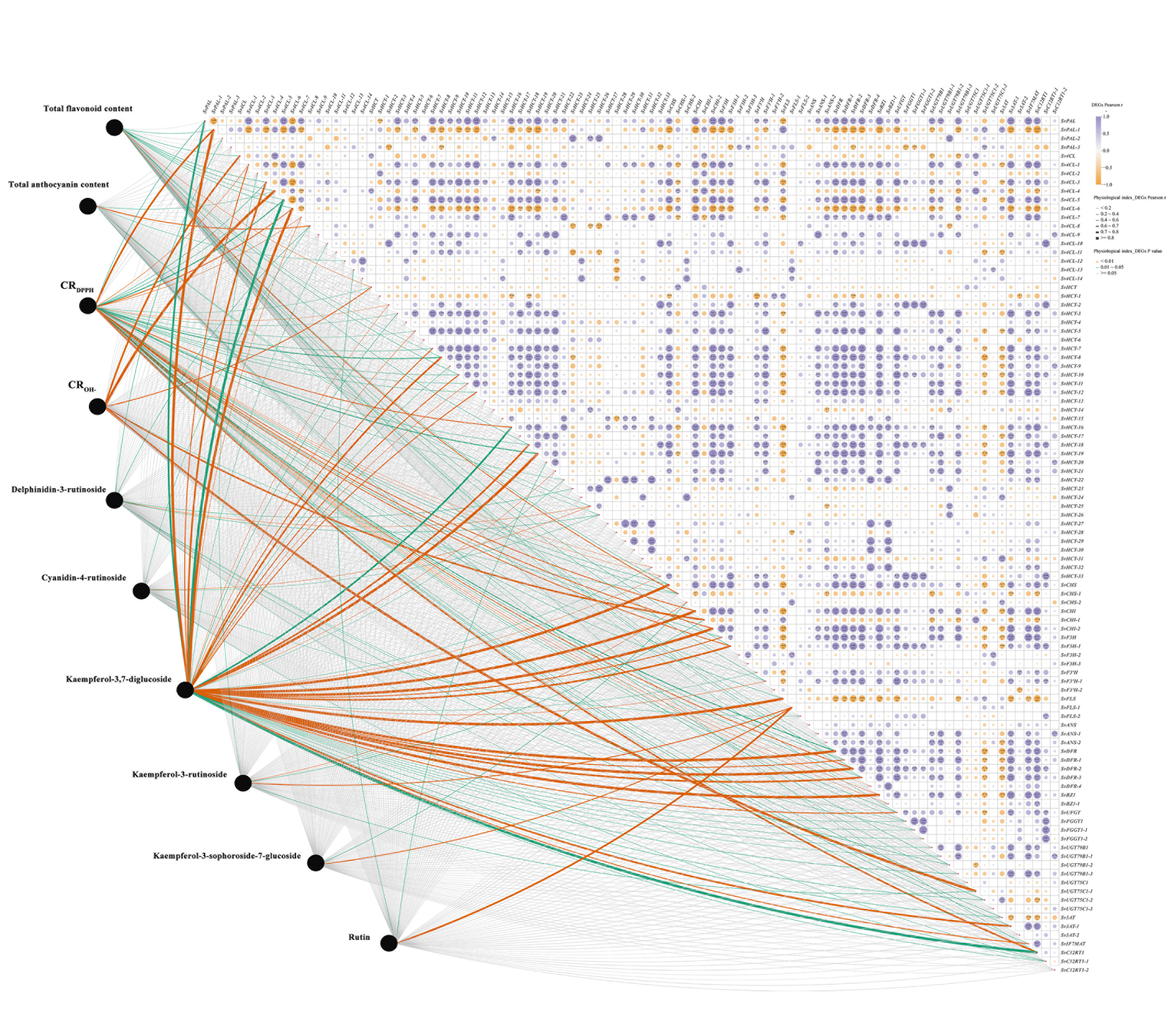

Supplement: Supplementary Figure 3 — Multi-omics association analysis in three cultivars of S. vulgaris. [file Image3.tif]
